# Intrinsically Multistable Soft Actuator Driven by Mixed‐Mode Snap‐Through Instabilities

**DOI:** 10.1002/advs.202307391

**Published:** 2024-03-06

**Authors:** Yichi Luo, Dinesh K. Patel, Zefang Li, Yafeng Hu, Hao Luo, Lining Yao, Carmel Majidi

**Affiliations:** ^1^ Department of Mechanical Engineering Carnegie Mellon University Pittsburgh PA 15213 USA; ^2^ Human‐Computer Interaction Institute, School of Computer Science Carnegie Mellon University Pittsburgh PA 15213 USA; ^3^ Department of Materials Science and Engineering Carnegie Mellon University Pittsburgh PA 15213 USA

**Keywords:** multistability, soft actuator, snap‐through instability

## Abstract

Actuators utilizing snap‐through instabilities are widely investigated for high‐performance fast actuators and shape reconfigurable structures owing to their rapid response and limited reliance on continuous energy input. However, prevailing approaches typically involve a combination of multiple bistable actuator units and achieving multistability within a single actuator unit still remains an open challenge. Here, a soft actuator is presented that uses shape memory alloy (SMA) and mixed‐mode elastic instabilities to achieve intrinsically multistable shape reconfiguration. The multistable actuator unit consists of six stable states, including two pure bending states and four bend‐twist states. The actuator is composed of a pre‐stretched elastic membrane placed between two elastomeric frames embedded with SMA coils. By controlling the sequence and duration of SMA activation, the actuator is capable of rapid transition between all six stable states within hundreds of milliseconds. Principles of energy minimization are used to identify actuation sequences for various types of stable state transitions. Bending and twisting angles corresponding to various prestretch ratios are recorded based on parameterizations of the actuator's geometry. To demonstrate its application in practical conditions, the multistable actuator is used to perform visual inspection in a confined space, light source tracking during photovoltaic energy harvesting, and agile crawling.

## Graphical abstract

In this work, a method is proposed to directly achieve intrinsic multistability within a single actuator unit. By employing this versatile multistable actuator, fast actuation across six stable states is presented within hundreds of milliseconds. Furthermore, potential applications of this shape‐reconfigurable actuator are presented for performing visual inspections, enhancing photovoltaic energy harvesting, and multimodal locomotion with combined crawling and turning.

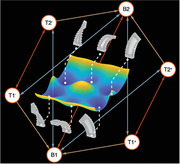

## 1 Introduction

Snap‐through instabilities have a central role in a wide range of bistable and multistable systems, from the venus flytrap^[^ ^1^ ^]^ to shape reconfigurable metamaterials^[^ ^2^ ^]^ and soft robot actuators.^[^ ^3^, ^4^ ^]^ For engineered systems, such structures can be designed to respond to a large variety of stimuli, including pneumatic/hydraulic pressure,^[^ ^5^, ^6^, ^7^, ^8^, ^9^, ^10^ ^]^ electric field,^[^ ^11^, ^12^, ^13^, ^14^ ^]^ magnetic field,^[^ ^15^, ^16^, ^17^, ^18^ ^]^ light,^[^ ^19^, ^20^, ^21^ ^]^ solvents,^[^ ^22^, ^23^, ^24^ ^]^ and humidity.^[^ ^21^, ^25^ ^]^ Bistable and multistable structures can be constructed from elastic beams,^[^ ^17^ ^]^ plates and shells,^[^ ^8^, ^12^, ^26^, ^27^, ^28^ ^]^ and thin‐walled balloons,^[^ ^5^, ^29^ ^]^ as well as from spring‐hinge structures^[^ ^30^, ^31^ ^]^ and stiffness‐tunable materials.^[^ ^32^ ^]^ They can also be patterned using origami,^[^ ^25^, ^33^, ^34^, ^35^, ^36^, ^37^, ^38^, ^39^ ^]^ kirigami,^[^ ^18^, ^40^ ^]^ and other metamaterial structures.^[^ ^2^, ^26^, ^41^ ^]^ Progress in the development of bistable and multistable structures has also led to a variety of breakthrough applications in the emerging field of soft robotics. These include mobile soft robots capable of rapid and multimodal locomotion,^[^ ^10^, ^31^, ^42^, ^43^, ^44^ ^]^ grasping and manipulation,^[^ ^6^, ^16^ ^]^ soft valves for controlling fluid flow,^[^ ^7^ ^]^ and soft logic devices.^[^ ^15^, ^25^, ^45^, ^46^ ^]^ Compared to traditional monostable counterparts, bistable, and multistable actuators embrace the following two unique advantages. First, snap‐through instability enables rapid response, as well as remarkable force output. After overcoming an initial energy barrier, the actuator automatically converges to a newly‐established stable state and can release a large amount of stored elastic potential energy. This can result in high‐performance actuators for fast locomotion^[^ ^10^, ^31^ ^]^ and jumping.^[^ ^8^, ^30^ ^]^ Second, once snapped, bistable and multistable actuators naturally maintain their configuration without the need for continuous energy input, something that is not viable in traditional monostable actuators. Consequently, it demonstrates a reliable solution for robot grasping,^[^ ^6^, ^16^ ^]^ dynamic changing in locomotion gait,^[^ ^43^ ^]^ structure morphing,^[^ ^32^, ^37^ ^]^ gated logic devices^[^ ^25^, ^45^, ^46^ ^]^ and electronics‐free‐control^[^ ^47^ ^]^ in a rapid and energy‐efficient fashion.

To further exploit the potential of snap‐through instability, researchers have endeavored to create multistable architectures that expand a system's total number of stable configurations and modes of deformation. This is typically achieved by directly combining multiple bistable units. For instance, researchers have proposed the creation of multistable origami arms capable of omnidirectional bending and twisting,^[^ ^16^ ^]^ multistable origami logic circuits,^[^ ^15^ ^]^ and multimodal deformation^[^ ^39^ ^]^ by serially connecting multiple units of Kresling origami bistable modules. Furthermore, a multistable soft flapping‐wing swimmer with enhanced maneuverability has been presented through the parallel combination of two pneumatic bistable actuators.^[^ ^10^ ^]^ Additionally, arrays of bistable shell units have been integrated to develop multistable thin‐walled domes^[^ ^26^ ^]^ and multistable responsive surfaces.^[^ ^24^ ^]^ Moreover, multistable metamaterials and structures typically require the spatial assembly of multiple unit cells in order to achieve more intricate shape changes.^[^ ^22^, ^41^ ^]^ Recently, researchers have combined kirigami with bistable dome structures, which leads to architectures with multiple stable states.^[^ ^18^, ^40^ ^]^ In addition, latching mechanisms and stiffness tuning have been adopted to develop multistable beams with latch‐able configurations.^[^ ^32^ ^]^ Although these precedents offer several feasible approaches for achieving multistable actuators, the direct realization of intrinsic multistability within a single actuator unit still remains a challenging task.

Here, we introduce an intrinsically multistable actuator unit that can adopt mixed‐mode snap‐through instabilities to realize six stable configurations, including two pure‐bend (B1 and B2) and four bend‐twist (T1^+^, T1^−^, T2^+^, and T2^−^) stable states. The actuator consists of a prestretched elastic membrane sandwiched between two elastomeric frames that are each embedded with a pair of shape memory alloy (SMA) coils. By precisely controlling the activation sequence and duration of the SMA coils individually, the actuator performs reversible transitions among its six stable states within a few hundred milliseconds. To examine this multistable response, we establish a comprehensive transition diagram and corresponding working principles for the three major types of stable state transitions. Additionally, we parameterize the actuator's geometry and empirically examine the influence of the prestretch ratio on the actuator's geometry and deformation. Finally, we present several demonstrations that highlight the ability to utilize this multistable actuator in various practical applications. The first demonstration is of an actuator mounted with a miniaturized camera for visual inspection. The second involves a heliotropism‐inspired energy‐harvesting that a photovoltaic cell is mounted on the actuator and tracks a moving light source to harvest energy. The third involves a multistable actuator with directionally asymmetric frictional feet that is capable of transitioning between crawling and turning motions.

## 2 Results

### 2.1 Multistable Actuator

**Figure 1:**
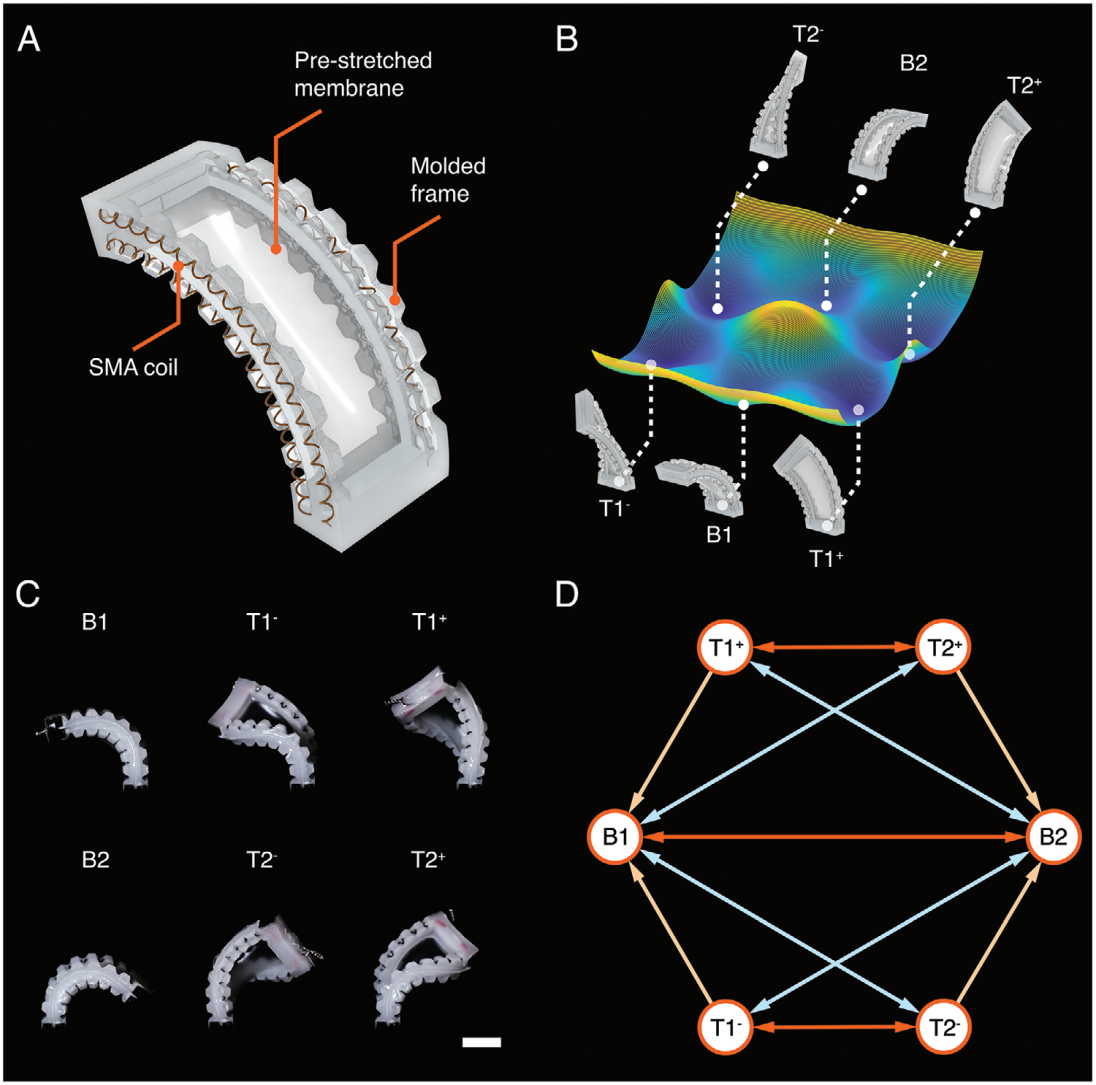
The multistable actuator. A) The overall structure of the multistable actuator including two injection‐molded frames with embedded SMA coils and a prestreched membrane. B) The schematic representation of the total elastic potential energy profile with six local minima, corresponding to six stable states: B1, T1^−^, T1^+^, B2, T2^−^, T2^+^, respectively. C) Real images of the multistable actuator with the prestretch ratio λ_*p*_ = 2 in all six stable states, (scale bar: 20 mm). D) The transition diagram of feasible transitions among all six stable states.

The multistable actuator consists of a prestretched silicone elastomeric membrane bonded between two injection‐molded silicone elastomeric frames that are each embedded with a pair of SMA coils (**Figure** **1**A). Adapted from a previous bistable actuator design,^[^ ^43^ ^]^ the dimensions and stiffness of the multistable actuator have been adjusted to enable torsional deformation of both sides of the frame. This torsional deformation mode allows for greater mixed‐mode interplay between the elastic potential energy of the prestretched membrane and the SMA‐embedded frames. Consequently, in addition to the previously established pure‐bend stable states, the multistable actuator also develops complex stable geometries that couple bending and twisting, which we henceforth refer to as “bend‐twist” states. The multistable actuator has six stable states in total, including two pure‐bend (B1 and B2) configurations and four bend‐twist (T1^+^, T1^‐^, T2^+^, and T2^‐^) configurations. Each of these corresponds to a local minimum on the total elastic potential energy manifold, as schematically represented in Figure 1B. The nomenclature of these stable states is based on the direction of bending and twisting: B1 (the pure‐bend stable state with a negative bending angle), T1^−^ (the bend‐twist stable state with a negative bending angle and a negative twisting angle), T1^+^ (the bend‐twist stable state with a negative bending angle and a positive twisting angle), B2 (the pure‐bend stable state with a positive bending angle), T2^−^ (the bend‐twist stable state with a positive bending angle and a negative twisting angle), T2^+^ (the bend‐twist stable state with a positive bending angle and a positive twisting angle), respectively. The real images of the multistable actuator in all six stable states are presented in Figure 1C. The actuator demonstrates the ability to rapidly transition among all six states with ease while maintaining stability without requiring any additional energy input (Movie S1, Supporting Information).

### 2.2 Stable States Transition

The reversible and rapid transition between stable configurations is controlled by the activation of the embedded SMA coils. Figure S4 (Supporting Information) depicts a multistable actuator in stable state B2, with four SMA coils named counterclockwise as SMA1, SMA2, SMA3, and SMA4, respectively. Through the precise control of activation timing for each SMA coil using individual transistors, diverse combinations of SMA activations can be achieved to realize transitions among the six stable states. As illustrated in Figure 1D, a transition diagram encompassing all possible transition routes has been proposed, exhibiting several transition capabilities. First, the multistable actuator is capable of transitioning from one pure‐bend state to another pure‐bend state with an opposite bending angle, e.g., from stable state B1 to B2 and B2 to B1. In addition, the multistable actuator is capable of transitioning between two bend‐twist states that share the same twisting direction with opposite bending directions, for example, between stable states T1^−^ and T2^−^. Moreover, transitions are achievable between a pure‐bend state and a bend‐twist state with opposite bending directions, such as between stable state B1 and T2^−^ or between B1 and T2^+^. It should be noted that direct transitions between bend‐twist states with different twisting directions, such as between stable state T2^+^ and T2^−^ or between stable state T1^+^ and T1^−^, are not possible in one transition (step) but it is viable in two steps as seen in the transition diagram (Figure 1D). The above transition is accomplished via a pure‐bend state (B1 or B2) which acts as a stable intermediate state or layover between two bend‐twist states with opposite twisting directions. For T2^+^ to T2^−^, first the actuator transitions to pure‐bend state (B1) and subsequently transitions from B1 to state T2^−^ and vice‐versa. Besides, the actuator is only able to transition back from a bend‐twist state to a pure‐bend state with the same bending direction, whereas the reverse transition is not directly feasible, e.g., feasible to directly transition from stable state T1^−^ to B1. In order to realize the reverse state transition from stable state B1 to T1^−^, triggering the actuator to T2^−^ and then further triggering it to T1^−^ is necessary (Movie S1, Supporting Information, from 12  to 20 s).

**Figure 2:**
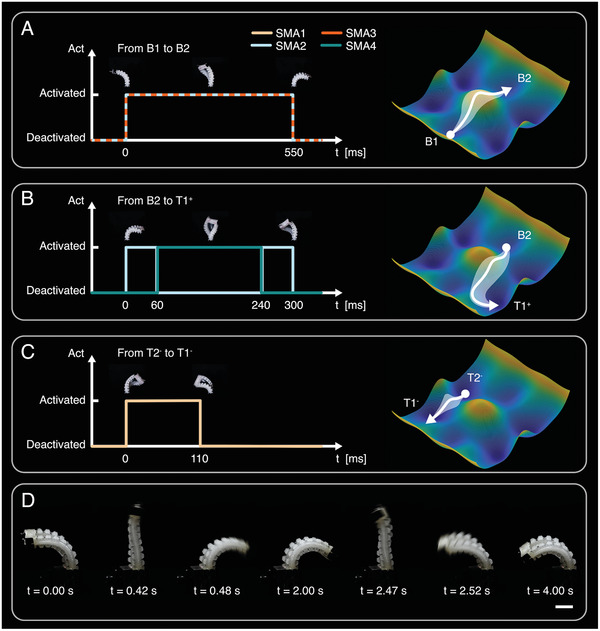
State transition of the multistable actuator. A) The activation sequence of SMA coils to transition from stable state B1 to B2 and corresponding transition route on schematic energy profile. B) The activation sequence for transitioning from stable state B2 to T1^+^. C) The activation sequence from stable state T2^−^ to T1^−^. D) Video snapshots showing the actuator (prestretch ratio λ_*p*_ = 2) snapping rapidly at a frequency of 0.5 Hz (Scale bar: 20 mm).

Next, three major types of transitions are selected for detailed examination. First, as depicted in **Figure** **2**A, the actuator can transition from one pure‐bend stable state B1 to another pure‐bend stable state B2. As the activation sequence of SMA coils demonstrated on the left, the transition is initiated by simultaneously activating SMA2 and SMA3 for a duration of 550 ms under a voltage of 15 V. This simultaneous activation ensures a symmetrical triggering of the actuator, which helps restrict potential torsional deformation and enables convergence to the desired pure‐bend stable state. The schematic energy profile on the right illustrates the idealized trajectory of the actuator's elastic potential energy during this transition. It is important to note that, due to the inherent fabrication errors in the manual manufacturing process, the actuator may not always exhibit perfect symmetry. This lack of strict symmetry could result in a slight deviation in the trajectory, represented by the translucent region surrounding the idealized trajectory.

Second, as a case of the transition from one pure‐bend stable state to another bend‐twist state with opposite bending directions, a transition from B2 to T1^+^ is presented in Figure 2B. The activation sequence shown on the left involves an initial activation of SMA2 for 60 ms, which temporarily stiffens the actuator asymmetrically and introduces a bias. Subsequently, SMA4 is activated for 180 ms, dragging the actuator in an asymmetric way. Above mentioned two asymmetric activations encourage torsional deformation, therefore the actuator flips to an opposite bending direction with its end twisted as well. Finally, SMA2 is activated for 60 ms again, preventing the actuator from overshooting to pure‐bend states B1, and ultimately converging to target bend‐twist state T1^+^. The idealized representation of the trajectory of the actuator's elastic potential energy during this transition is illustrated on the right. In all of these cases, SMA activation is achieved by applying 15 V of voltage.

Lastly, as an example of transitions between bend‐twist states with the same twisting directions, Figure 2C demonstrates the transition from the stable state T2^−^ to T1^−^. In this case, SMA1 is activated for 110 ms under a 15 V voltage, triggering the actuator to transition while maintaining the twisted configuration. The idealized representation of the actuator's trajectory of elastic potential energy during this transition is depicted on the right.

In addition to the sequence and duration of SMA activation for various state transitions, we also examine the actuator cool‐down time *t*_*c*_ between transitions. It should be noted that the state transition between two pure‐bend states B1 and B2 requires the longest activation time, therefore represents the most extreme case for heat dissipation within the three major types of transition. Therefore, we tested the maximum feasible frequency of state transition between B1 and B2. As shown in Figure 2D, the actuator can perform repeated cycles of actuation at a frequency of 0.5 Hz when transitioning between states B1 and B2, which corresponds to an activation time *t*_*a*_ = 550 ms and cooling time *t*_*c*_ = 1450 ms. Such a frequency is repeated for five cycles of transition as shown in Supporting Information, Movie S2 (Supporting Information). The corresponding plot of the sequence and duration for SMA activation and cooling at 0.5 Hz can be found in Figure S5 (Supporting Information). It should be noted that *t*_*c*_ is primarily for heat‐dissipation and therefore related to λ_*p*_. With smaller λ_*p*_, the energy barrier for snap‐through is smaller, resulting in a shorter *t*_*a*_. In this case, *t*_*c*_ could also be shortened to achieve higher frequency.

### 2.3 Characterization of Stable States

**Figure 3:**
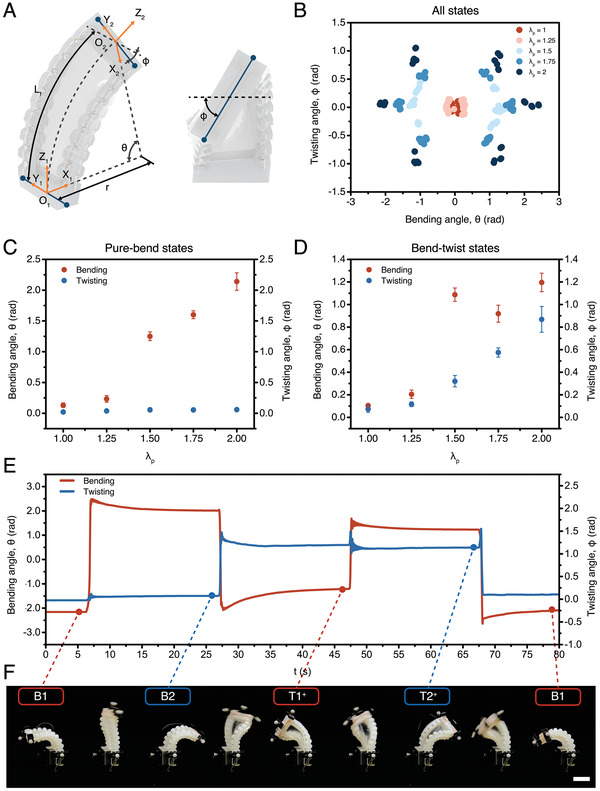
Stable states characterization of the multistable actuator. A) Parameterization of the actuator's geometry. B) The distribution of six stable states along prestretch ratio λ_p_ varying from 1 to 2. C) The changes of bending angle θ and twisting angle ϕ for pure‐bend states when the prestretch ratio λ_p_ varies from 1 to 2. D) The changes of bending angle θ and twisting angle ϕ for bend‐twist states with prestretch ratio λ_p_ varying from 1 to 2. E) The changes in bending angle θ and twisting angle ϕ of a multistable actuator (prestretch ratio λ_*p*_ = 2) in successive stable state transitions and F) corresponding snapshot images from Movie S3 (Supporting Information) (scale bar: 25 mm).

To further investigate the actuator's geometry, we parameterized the actuator's configuration with two parameters: bending angle θ and twisting angle ϕ. As illustrated in **Figure** **3**A, the actuator is depicted as first bending along axis Y_1_ with a bending angle θ and subsequently twisting along axis Z_2_ with a twisting angle ϕ. Since the length of the centerline of the frame is assumed to be a constant L_f_, the bending radius r can be estimated as *r* = *L*_f_/θ. Since the two ends of the frame are significantly stiffer than the bending portion, the width of the two ends of the frame can also be assumed as a constant W_f_. Therefore, the relationship between the global position of the four edges of the frame and two configuration parameters (θ, ϕ) is established. Detailed calculations could be found in Supporting Information.

Next, a motion capture system is employed to capture and record the actuator's shape both in its stable configurations and during transitions. A detailed description of the motion capture system and marker distributions could be found in Figure S7 (Supporting Information). Actuators with prestretch ratios λ_p_ varying from 1 to 2 are tested, with a step size of 0.25. For each specific prestretch ratio, three different actuators are examined and repeated three times on each stable state. As the overall result shown in Figure 3B, when λ_p_ is relatively small (e.g., λ_p_ ⩽ 1.25), the elastic potential energy stored within the prestretched membrane is insufficient to establish stable states. When the prestretch ratio λ_p_ increases to 1.5, the actuator starts to exhibit pure‐bend stable states while the bend‐twist states are not highly distinguishable from pure‐bend states. Finally, when the prestretch ratio λ_p_ is large enough, (e.g., λ_p_ ⩾ 1.75), bend‐twist stable states start to deviate from pure‐bend states, which suggests each stable state is more distinguishable and stable.

Furthermore, we study the case of pure‐bend and bend‐twist states individually. Figure 3C shows the absolute value of bending angle θ and twisting angle ϕ as a function of the prestretch ratio λ_p_ of the elastic membrane for pure‐bend stable states. It is observed that when λ_p_ increases from 1 to 2, the bending angle θ increases from 0.13  to 2.14 rad, while the twisting angle ϕ remains small (0.02 rad ⩽ ϕ ⩽ 0.06 rad), and the bending angle exhibits a significant increase between λ_p_ = 1.25 and λ_p_ = 1.5. On the other hand, as presented in Figure 3D, the bending angle of bend‐twist states also shows a big increase when λ_p_ reaches 1.5 and remains within a stable range with a relatively small increase rate. Additionally, the twisting angle ϕ of bend‐twist states starts to continuously increase from 0.32  to 0.86 rad when λ_p_ increases from 1.5 to 2.

Lastly, the changes in bending angle θ and twisting angle ϕ in successive transitions between stable configurations are recorded as well (Figure 3E). The actuator is initialized in stable state B1, with a bending angle θ = ‐2.16 rad and a twisting angle ϕ = ‐0.02 rad. At t ≈ 7 s, SMA2 and SMA3 are activated for 550 ms, triggering the actuator to transition to another pure‐bend stable state B2, with a bending angle θ = 2.05 rad and a twisting angle ϕ = 0.08 rad. Subsequently, at t ≈ 27 s, SMA2, SMA4, and SMA2 are activated sequentially for 60, 180, and 60 ms, respectively, to trigger the actuator deforming to bend‐twist stable state T1^+^, with a bending angle θ = ‐1.21 rad and a twisting angle ϕ = 1.19 rad. Next, at t ≈ 47 s, SMA2 is activated for 110 ms, enabling the actuator to transition to another bend‐twist stable state T2^+^ with a bending angle θ = 1.23 rad and a twisting angle ϕ = 1.14 rad. Ultimately, SMA1 and SMA4 are activated simultaneously for 300 ms so that the actuator reconfigures to the initial pure‐bend stable state B1 with a bending angle θ = ‐2.07 rad and a twisting angle ϕ = 0.11 rad. The corresponding images of each stable state and the entire transition are shown in Figure 3F and Movie S3 (Supporting Information). All the above‐mentioned SMA activation is performed with 15 V of applied voltage. This successive transition also provides support to the trajectory illustrated in our idealized representation of the elastic potential energy profile. When the actuator transitions from one pure‐bend state (i.e., B1) to the opposite pure‐bend stable state (i.e., B2) the twisting angle remains almost 0 and the change of the bending angle is dominant (Figure 2A). Additionally, when the actuator is triggered from one pure‐bend state (i.e., B2) to a bend‐twist with an opposite bending direction (i.e., T1^+^), the bending and twisting angle both change dramatically (Figure 2B). Finally, when the actuator switches from one bend‐twist state (i.e., T1^+^) to the opposite bend‐twist state with the same twisting direction (i.e., T2^+^), the twisting angle remains stable while the bending angle flips (Figure 2C).

### 2.4 Demonstrations

In order to broaden our understanding of the potential applications of the multistable actuator, three different cases are presented here. The first employs a multistable actuator with a miniature camera mounted on its tip to perform visual inspections in a confined space. The second is a demonstration of heliotropism‐inspired energy harvesting in which a photovoltaic cell is mounted to the tip of an actuator that tracks the changing position of a moving light source. Lastly, a dexterously‐turning crawler is demonstrated in which footpads with anisotropic friction are mounted to the actuator's front and rear ends.

#### 2.4.1 Visual Inspection with a Tip‐Mounted Camera

**Figure 4:**
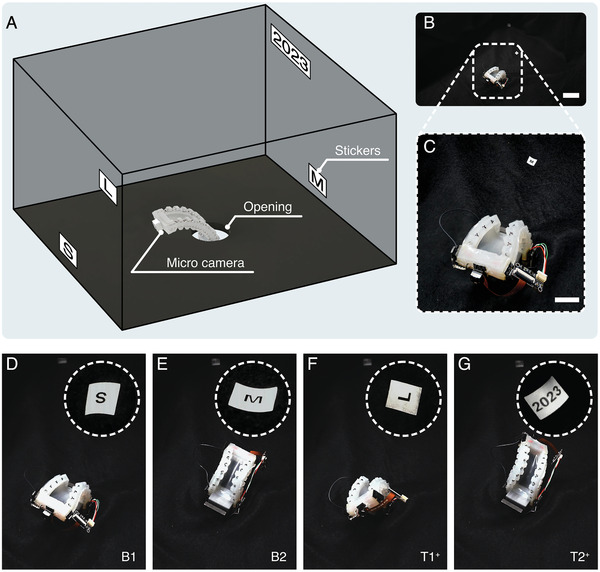
Visual inspection in a confined space using a multistable actuator with a tip‐mounted camera. A) Schematic of the working environment within the confined space. B) A multistable actuator for confined space visual exploration with a miniature camera mounted on its tip (scale bar: 40 mm). C) A zoomed‐in image of the multistable actuator (scale bar: 20 mm). D) The multistable actuator in stable state B1, with the miniature camera capturing the letter “S”. E) The multistable actuator in stable state B2 where the miniature camera captures the letter “M”. F) The multistable actuator in stable state T1^+^ with the camera's view showing the letter “L”. G) The multistable actuator remains in stable state T2^+^, enabling the miniature camera to record the number “2023”, forming an entire string of “SML2023”.

The multistable actuator is inserted in a box through a narrow opening on its bottom wall, and a miniature camera is mounted on the tip of the actuator to enable visual inspection within the box (see **Figure** **4**A; Movie S4, Supporting Information). Inside the box are four stickers bearing different letters and numbers that are attached to distinct locations along the inner walls. Figure 4B, C present photographs of the actuator within the confined space. To orient the camera toward each of the stickers, the actuator is triggered to transition between different stable states. Initially, as illustrated in Figure 4D, the actuator stays in state B1, and the miniature camera on the tip captures the first letter “S” as indicated by the dashed circle. Then, SMA2 and SMA3 are activated simultaneously for 550 ms to trigger the actuator to transition from stable state B1 to B2, where the view of the miniature camera (shown in the dashed circle in Figure 4E) captures the second letter, “M”. Next, a sequential activation process is employed to guide the actuator through a specific trajectory. We first activate SMA2 for 60 ms to introduce a temporary stiffness bias to the structure, and then activate SMA4 for 180 ms to let the actuator overcome the energy barrier beyond an asymmetric route, subsequently, SMA2 is activated for 60 ms again to ensure convergence to the stable state T1^+^, where the miniature camera captures the third letter, “L”, as shown in the dashed circle in Figure 4F. Finally, SMA2 is activated for 110 ms, facilitating the transition of the actuator from stable state T1^+^ to T2^+^, where the miniature camera records the number “2023” (depicted within the dashed circle in Figure 4G). As a result of successfully exploring the confined space, the captured letters and numbers can be arranged to form the string “SML2023”. It is important to note that, apart from its exploration capabilities within the internal environment of a confined space, the actuator can also maintain a specific stable state for the purpose of continuously monitoring multiple locations without requiring additional energy input, owing to its inherent stability.

#### 2.4.2 Heliotropism‐Inspired Energy Harvesting

Heliotropism pertains to a phototropic response observed in certain plant species, notably the sunflower (*Helianthus annuus*).^[^ ^48^ ^]^ It involves the dynamic movement of the plant stem, specifically the reorientation of their shoot apices, in accordance with the sun's apparent movement across the sky throughout the day. At dawn, these plants orient their shoot apices towards the east, and by dusk, they adjust their orientation to face west. This rhythmic adjustment allows these plants to optimize their photosynthesis efficiency, thereby maximizing their energy acquisition from solar radiation.^[^ ^48^ ^]^

**Figure 5:**
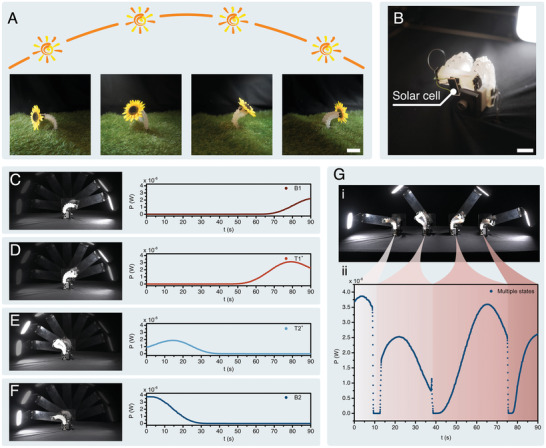
Heliotropism‐inspired energy harvesting based on the multistable actuator. A) The schematic view of efficient energy harvesting inspired the heliotropism phenomenon of sunflowers (scale bar: 40 mm). B) A miniature photovoltaic cell mounted on the tip of the multistable actuator for energy harvesting (scale bar: 20 mm). C) Harvesting energy when the actuator remains in a single stable configuration B1, with the corresponding output power generated by the photovoltaic cell with respect to time. D) Power output over time for an actuator that remains in the stable configuration T1^+^. E) Power output for a configuration T2^+^. F) Power output for a configuration B2. G) Power output over time as the actuator is actively transitioned between various stable states in order to track the position of the moving light source.

Taking inspiration from heliotropism, we have employed a multistable actuator as a means to enhance the energy harvesting output of a photovoltaic cell (**Figure** **5**A; Movie S5, Supporting Information). Our experimental setup involves mounting a miniature photovoltaic cell to the tip of the actuator, allowing it to capture energy emitted by an LED (Figure 5B; Figure S13, Supporting Information). To simulate the sun's movement, the LED is attached to an acrylic limb driven by a servo motor, causing it to move in a circular path with a cycle time of t = 90 s. The photovoltaic cell is serially connected to a resistor as a power supply and the voltage applied to the resistor is measured to calculate the output power and calculation can be found in Supporting Information. Initially, the output power of the photovoltaic cell with a single stable state has been examined. When the actuator remains in stable state B1, the energy output is initially close to zero, but it begins to increase after t > 70 s, reaching its peak at t ≈ 90 s when the LED is positioned at a similar altitude to the photovoltaic cell (Figure 5C). In the case of state T1^+^ (see Figure 5D), the energy output reaches its maximum around t ≈ 75 s, which is much earlier than stable state B1, corresponding to an LED altitude angle of approximately 45°. Similarly, as illustrated in Figure 5E, the energy output reaches its maximum at t ≈ 15 s when the actuator maintains steady in stable state T2^+^. Finally, for stable states B2 (illustrated in Figure 5F), initially the energy output is large but then subsequently decreases when the LED rises and ultimately converges to 0 at t ≈ 30 s. Consequently, in the presence of a single stable state, where the actuator maintains a static configuration, the photovoltaic cell can only achieve high output power within a limited range of altitude angles. The average output power at each single stable state are 2.74× 10^−7^ W (B1), 8.20× 10^−7^ W (T1^+^), 4.83× 10^−7^ W (T2^+^), and 6.58× 10^−7^ W (B2), respectively.

Next, in order to enhance the output power for the entire duration of the light source's motion, the multistable actuator is activated to transition between its various stable configurations (Figure 5G). As the LED starts to rise, the actuator is initialized in stable state B2, resulting in high energy output at the beginning of the LED cycle. When the output power starts to drop, a transition to stable state T2^+^ is necessary. However, it should be noted that the direct transition from B2 to T2^+^ is not feasible according to our previously proposed transition diagram (see Figure 1D), as the energy required for such state transition is below the minimum activation energy for phase changing of SMA coils. Hence, in the experiment, we first activate SMA4 for 60 ms for temporary asymmetric stiffening, and then activate SMA2 for 180 ms for overcoming the energy barrier, and subsequentially activate SMA4 for 60 ms again for convergence to stable state T1^+^. Ultimately, after cooling for *t*_*c*_ = 2000 ms, SMA4 is activated for 110 ms to enable a transition from stable state T1^+^ to T2^+^. This sequence of transitions enables the output power of the photovoltaic cell to remain high in the new stable state T2^+^. When the LED reaches the midpoint of its trajectory at t ≈ 45 s, where the stable state T2^+^ exhibits low power output, it is triggered to transition to T1^+^ via activating SMA2 for 110 ms, resulting in a gradual increase in power output. Finally, as the LED's altitude becomes significantly lower and the power output of the photovoltaic cell drops again, the actuator transitions from state T1^+^ to B1, where the power output increases again and remains high until the LED reaches the end of its trajectory. The average output power of the photovoltaic cell when utilizing multiple stable states is 2.24× 10^−6^ W. Compared to the cases with single stable states, the output power is improved by 637.4% w.r.t. state B1, 207.5% w.r.t. state T1^+^, 146.6% w.r.t. state T2^+^, and 318.6% w.r.t. state B2, respectively.

Hence, by utilizing multiple stable states sequentially (B2, T2^+^, T1^+^, B1, respectively), the photovoltaic cell maintains a consistently high output power as we move the LED to mimic the sun's trajectory throughout the day. The resulting plot in Figure 5G ii exhibits four distinct peaks, each corresponding to the peak output power of the respective stable configurations. Notably, the actuator's transition time is in the range of hundreds of milliseconds, significantly shorter than the timescale of a day. Since the actuator can maintain stability within a stable state without requiring additional power input, it offers an energy‐efficient approach to enhance the power output from the photovoltaic cell.

#### 2.4.3 Dexterously‐Turning Crawler

**Figure 6:**
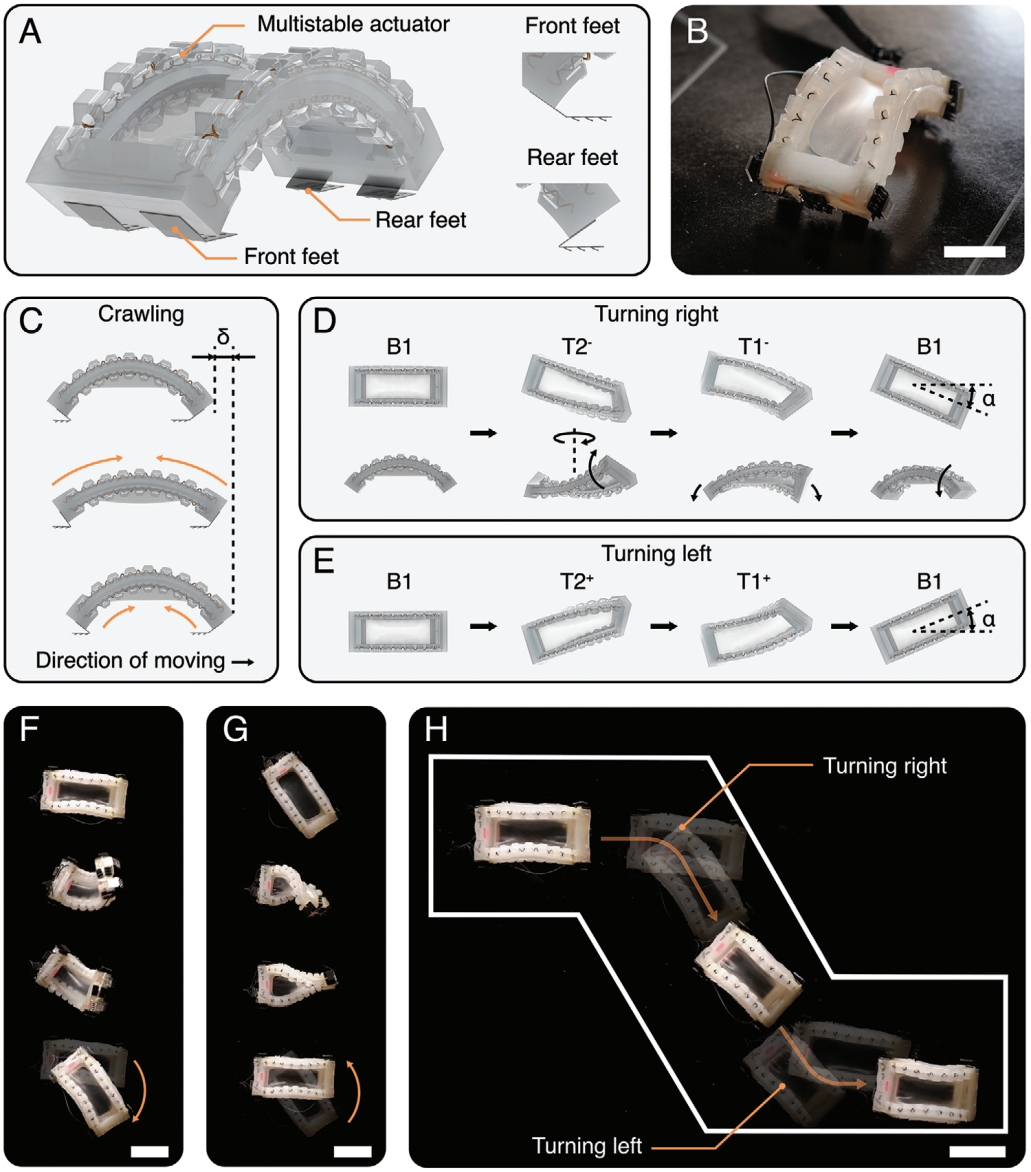
A dexterously‐turning multimodal crawler based on the multistable actuator. A) The design of the crawler by attaching two pairs of directionally asymmetric friction feet to the multistable actuator. B) The real image of the crawler (Scale bar: 20 mm). C) The gait plot of crawling forward. The gait plot of turning right (D) and turning left(E), respectively. The snapshots of the crawler turning right (F) and turning left(G), respectively (Scale bar: 40 mm). H) The demonstration of the crawler accomplishing a z‐shape zig‐zag trajectory (Scale bar: 40 mm).

The multistable actuator is also capable of achieving agile multimodal locomotion on its own without reliance on additional actuators. To demonstrate this, we have implemented the actuator as a dexterous crawler capable of both crawling and turning. As depicted in **Figure** **6**A,B the crawler consists of a single actuator with two pairs of laser‐cut footpads attached to its front and rear feet ends. These feet are designed with arrays of tilted teeth, which impart directionally asymmetric friction, enabling movement in one direction. As the crawling gait in Figure 6C shows, the crawler is initially in stable state B1. To initiate crawling, both SMA2 and SMA3 are simultaneously activated for *t*_*a*_ = 300 ms. Due to the gravitational force and the friction, such activation doesn't flip the actuator to an opposite stable state and instead, the actuator becomes temporarily flattened. As a result, the actuator's curvature decreases, causing an increase in the distance between the feet. Owing to the directionally asymmetric friction on the feet, the tip of the actuator moves forward while the end remains fixed. After a cooling period of *t*_*c*_ = 3000 ms, SMA1 and SMA4 are simultaneously activated for *t*_*a*_ = 300 ms, causing the actuator to return to its initial pure‐bend state B1. In this case, the tip of the actuator remains fixed while the end moves forward, facilitating crawling. All the above‐mentioned activation steps are performed with an applied voltage of under a voltage of 15 V. The average length of each stride is measured as δ = 25.6 ± 3.6 mm. The corresponding detailed plot of the sequence and duration for SMA activation and cooling is presented in Figure S14 (Supporting Information).

In order to execute turning motions, the crawler utilizes bend‐twist states. To generate a right turn (Figure 6D), SMA3 is activated for *t*_*a*_ = 250 ms, inducing the actuator to transition into a bend‐twist state T2^−^, followed by a cool‐down time of *t*_*c*_ = 1500 ms. Subsequently, SMA1 is activated for *t*_*a*_ = 250 ms, with a cool down time of *t*_*c*_ = 3000 ms, driving the actuator toward the opposite bend‐twist state T1^−^. By utilizing two bend‐twist states with negative twisting angles, a rotational force is generated, enabling the crawler to turn right. Finally, the actuator returns to the pure‐bend state B1 by activating SMA1 and SMA4 for *t*_*a*_ = 250 ms. In Figure 6D, the upper row illustrates the top view during the turn while the lower row shows the side view. Similarly, a left turn is achieved by employing the state‐transition sequence of B1, T2^+^, T1^+^, and B1 (Figure 6E). All of the above‐mentioned SMA activations are performed with 15 V of applied voltage. The average turning angle is measured as α = 0.77 ± 0.17 rad. The detailed descriptions of the SMA activation and cooling for gait control are shown in Figures S15 and S16 (Supporting Information). The corresponding snapshots of turning right and left are shown in Figure 6F, G, respectively.

Finally, we demonstrate the crawler's ability to move along a z‐shaped zig‐zag pathway (Figure 6H, and Movie S6, Supporting Information. The crawler accomplishes this trajectory through a combination of crawling and turning gaits. In this way, we show agile multimodal locomotion using only a single actuator unit.

## 3 Discussion and Conclusion

This paper reports a soft actuator that is intrinsically multistable and capable of rapid transition between various configurations through the electrical activation of embedded nitinol coils. The actuator is composed of two elastomeric frames that are embedded with a pair of nitinol SMA coils and which are bonded to opposite sides of a prestretched elastic membrane. The actuator has six stable states: two pure‐bend states, named B1 and B2, and four bend‐twist states, named T1^+^, T2^+^, T1^−^, and T2^−^. Thanks to the combination of mixed‐mode snap‐through and SMA activation, the actuator is capable of rapid transitions among all six stable states at speeds on the order of hundreds of milliseconds. Among the various possible transitions, three representative types are highlighted in this work: i) transitions from a pure‐bend stable state to an opposing pure‐bend stable state; ii) transitions from a pure‐bend stable state to a bend‐twist state that manifests an opposite bending direction; iii) transitions from one bend‐twist state to another bend‐twist state, maintaining the same twist direction while modifying the bending direction to the opposite. By employing this control strategy, the system can effectively navigate between different stable states, enabling versatile and agile behavior. Furthermore, the actuator's geometry is parameterized, allowing for the measurement of documentation of bending and twisting angles in all stable states across different prestretch ratios. This parameterization facilitates the characterization of transitional behavior as well. A series of practical implementations are presented to demonstrate the potential applications of the multistable actuator. These applications encompass the visual inspection of confined spaces, the enhancement of energy harvesting based on principles of heliotropism, and a multimodal crawler with agile locomotion capabilities. By utilizing a single unit of this multistate actuator, rapid transitions and morphing can be achieved without the need for complex designs with multiple bistable actuator units.

While promising, there are still areas in which the multistable actuator can be better understood or improved. First, the size of our multistable actuator is limited to the centimeter scale due to constraints imposed by the use of nitinol coils. In principle, it is possible to use other actuator technologies or to create a structure that is fully passive, as has been demonstrated in Movie S7 (Supporting Information) in which SMA is replaced with 3D‐printed elastic rods (Flexible 80A, Formlabs Inc.). The key element in our approach lies in providing multiple routes (bending and twisting) for the system to fulfill the “desire” of the prestretched membrane to contract. Consequently, the dimensions and geometry of the actuator can be freely scaled, while the actuation method should be modified correspondingly. For example, the actuator could potentially be reduced to the millimeter scale and triggered by the magnetic field.

Moreover, the elastic potential energy profile shown in Figure 1B is just a schematic representation, while the theoretical computation of the potential energy still remains unsolved. While an analytic model has been proposed in Supporting Information, it is currently limited to predicting two pure‐bend stable states. This limitation arises from two key factors: i) The deformation of the frame in bend‐twist modes exhibits considerable complexity, surpassing the capabilities of our current analytic model in accurately assuming its geometry; ii) The prestretched membrane has a tendency to contract not only along its length but also its width when the structure bends, which causes the center of the frames to twist inwards – a deformation that is not currently accounted for in the analytic model. To overcome these limitations, a more complete computational simulation that accounts for the entirety of the nonlinear 3D deformation of the frames, coils, and membrane is required. One possible future research direction is using finite element analysis (FEA) to simulate the multistable actuator. However, to carefully and precisely simulate this complex structure, there are a few points of challenge to overcome. First, the frame consists of multiple materials (silicone elastomer and SMA coils) and has a complicated shape (channel for SMA coils and wavy surface pattern). This characteristic will result in discontinuity and nonlinearity of the system and would be computationally expensive to simulate. In addition, the mixed‐mode snap‐through involves both twisting and bending deformation, thus the correct convergence of the stable states is challenging. Moreover, the relaxation of the prestretched elastic membrane might also require consideration.

Additionally, the merit of bistable and multistable actuators lies in their ability to undergo rapid transitions between stable states while maintaining stability without the need for additional energy input for control. However, the presence of external forces can impact the stable states, and in extreme occasions, the stable states can be lost. This could impede precise manipulation and interaction in practical applications. One potential solution is to employ a hybrid approach that incorporates snap‐through instability for large‐scale transitions, while locally controlling the system around the desired stable states when external forces are involved. Another possible approach is utilizing stiffness‐tuning and latching mechanisms to lock and secure the stable state.^[^ ^32^ ^]^ By adjusting the stiffness of specific components to latch the actuator in a particular stable configuration, the actuator's stability and response to external forces could be improved.

## 4 Experimental Section

### Actuator Fabrication

The multistable actuator was fabricated through three major steps: i) Fabricating the elastomeric frame by injection molding; ii) fabricating the elastic membrane by utilizing a thin‐film applicator; iii) Prestretching the membrane on a stretcher and carefully aligning and attaching frames to both sides of the membrane. A detailed description of the entire fabrication process can be found in Figures S1–S3 (Supporting Information).

### Stable States Characterization

The stable states of the multistable actuator with different prestretch ratios were characterized based on a motion capture system (Motive, OptiTrack). Eight markers were attached to the platform of the actuator to establish global coordinates and local information. A detailed explanation of the mocap system and distribution of markers are included in Figure S7 (Supporting Information).

### Confined‐Space Exploration

A miniature camera (5MP Camera Module, Qinlorgo) was mounted to the tip of the actuator (Figure 4B) via dual lock tapes (Scotch Self‐Mating Reclosable Fastener, REBUILD SKILLS). The view of the miniature camera was transferred to a laptop via a USB cable.

### Heliotropism‐Inspired Energy Harvesting

A miniature photovoltaic cell (KXOB25‐14X1F‐TB, ANYSOLAR Ltd.) was mounted on the tip of the actuator (Figure 5B) for energy harvesting via dual lock tapes (Scotch Self‐Mating Reclosable Fastener, REBUILD SKILLS). The full description of the experimental setup is provided in Figure S11 (Supporting Information).

### Dexterously‐Turning Crawler

The directionally asymmetric friction feet were fabricated using stainless steel shim (thickness 55 µm) adopting a UV laser cutter (LPKF ProtoLaser U3 laser system). After laser cutting the 2D pattern, the triangular teeth on the feet were manually curved by tweezers, then attached to the multistable actuator using super glue. A detailed explanation of the gait control for crawling forward and turning directions was provided in Figure S12–S14 (Supporting Information).

## Conflict of Interest

The authors declare no conflict of interest.

## Supporting information

Supporting Information

Supplemental Video 1

Supplemental Video 2

Supplemental Video 3

Supplemental Video 4

Supplemental Video 5

Supplemental Video 6

Supplemental Video 7

## Data Availability

The data that support the findings of this study are available in the supplementary material of this article.
