# Supplementary material for: Intrinsically Multistable Soft Actuator Driven by Mixed‐Mode Snap‐Through Instabilities
Source: Adv Sci (Weinh). 2024 Mar 6;11(18):2307391. doi: 10.1002/advs.202307391 (PMC11095224; doi:10.1002/advs.202307391)
Supplement: Supplementary file 1 — Supporting Information [file ADVS-11-2307391-s006.pdf]

ADVANCED SCIENCE
Open Access

Supporting Information

for *Adv. Sci.*, DOI 10.1002/advs.202307391

Intrinsically Multistable Soft Actuator Driven by Mixed-Mode Snap-Through Instabilities

Yichi Luo, Dinesh K. Patel, Zefang Li*, Yafeng Hu, Hao Luo, Lining Yao and Carmel Majidi**

Supporting Information

Intrinsically multistable soft actuator driven by mixed-mode snap-through instabilities

*Yichi Luo, Dinesh K. Patel, Zefang Li, Yafeng Hu, Hao Luo, Lining Yao, Carmel Majidi**

Frame fabrication

The frame of the multistable actuator is fabricated via injection molding (Fig. S1). The molds for injection molding include four 3D printed parts as shown in Fig. S1*A* (Form 3+, Formlabs Inc.). Six nuts (18-8 Stainless Steel Flange Nut, 93033A107, McMaster-Carr) are installed into mold part 1 for alignment. Two metallic rods (Hardened Oversized High-Speed M2 Tool Steel Rod, 3023A338, McMaster-Carr, cut into a length of 90 mm) are inserted into the holes on Part 3 and 4 to create tunnels for inserting SMA coils after the injection molding process. After assembling the parts and the rods, six screws (18-8 Stainless Steel Socket Head Screw, 91292A029, McMaster-Carr) are adopted for further aligning and tightening molds (Fig. S1*B*). It is recommended to use C-clamps to clamp the molds as well to avoid possible deformation in the oven. Next, Part A and Part B of silicone elastomer (Dragon-skin 30, Smooth-on, Inc.) are filled into two syringes connected with a mixing nozzle at the end. Subsequently, air pressure (90 kPa) will be applied on the piston of each syringe to conduct injection molding, forcing two parts of silicone elastomer to mix thoroughly in the mixing nozzle and then injected into the mold and the excessive materials will flow out from the ejection pins on the sides of the frame (Fig. S1*C*). Degassing Part A and Part B within their syringe before injection would significantly improve the quality of the injection. Then, a plug is inserted into the injection hole to prevent the silicone elastomer from back-flowing due to the temperature rise after putting the molds into the oven (Fig. S1*D*). The mold and the injected elastomer are cured in the oven under 70 °C for 6 hours (Fig. S1*E*). After fully curing, the frame is brought out from the mold for further assembly (Fig. S1*F*).

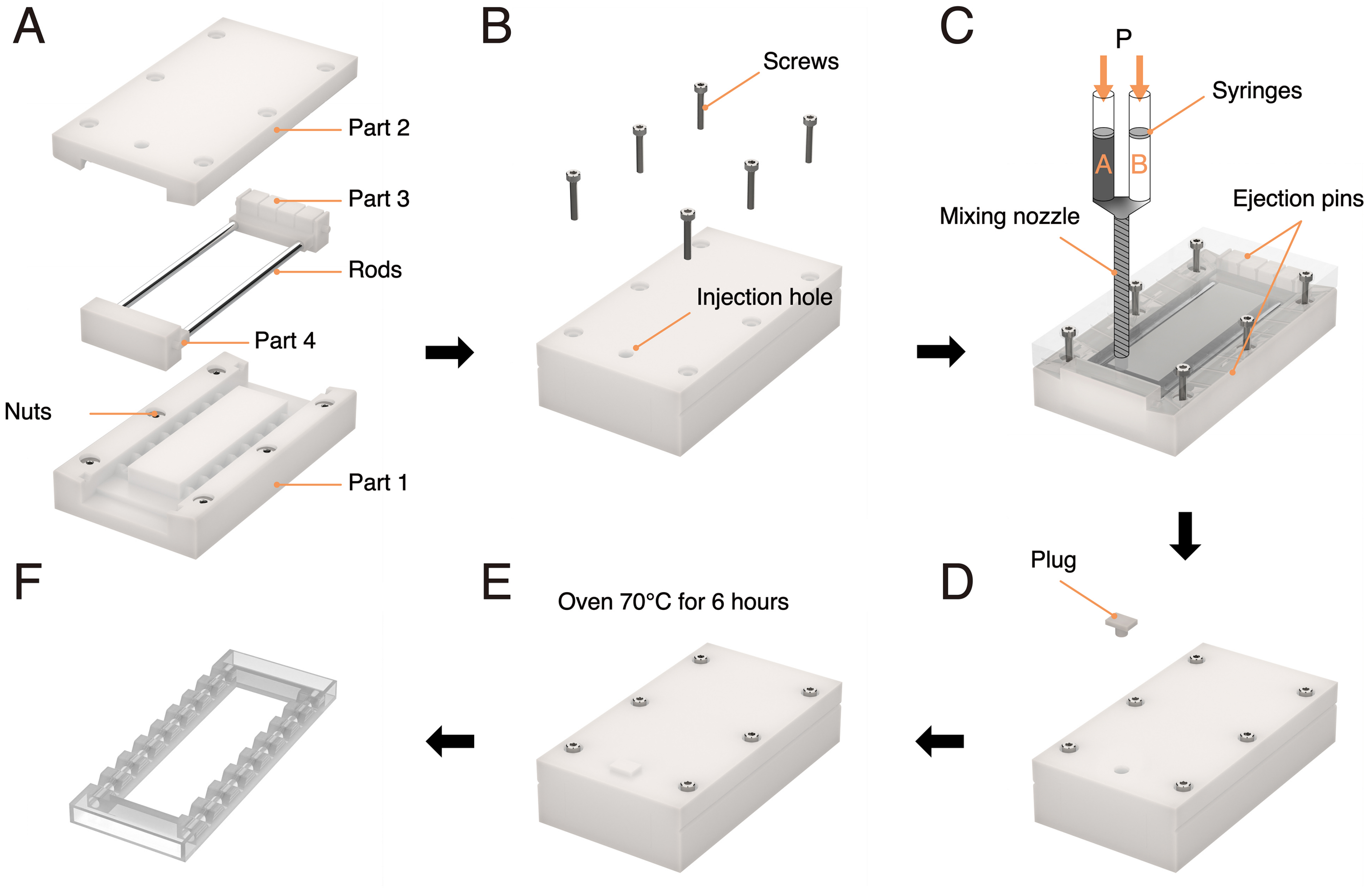

Figure S1: Frame fabrication process. (*A*) Four parts of the mold. (*B*) Screwing the assembled mold for alignment. (*C*) Injection molding. (*D*) Inserting the plug into the injection hole. (*E*) Oven curing. (*F*) Finishing fabrication of the frame.

Membrane fabrication

The fabrication process for the elastic membrane is illustrated in Fig. S2. First, the same weight amount of Part A and Part B of silicone elastomer (Dragon-skin 30, Smooth-on, Inc.) are fixed together and shear mixed in a planetary mixer (AR-100, Thinky Corporation) for 30 s and then degassed for 10 min (Fig. S2*A*). Then, after the elastomer is poured onto the surface of a smooth aluminum sheet (Fig. S2*B*), a thin-film applicator is employed to form a thin layer of elastomer (Fig. S2*C*). Then the thin layer of membrane is cured in the oven under 70 °C for 3 hours. After fully curing, a CO_2 laser machine (30 W VLS 3.50, Universal Laser Systems) is utilized to cut the membrane into a rectangular shape (length 190 mm, width 120 mm) for further membrane stretching and actuator assembly (Fig. S2*D*).

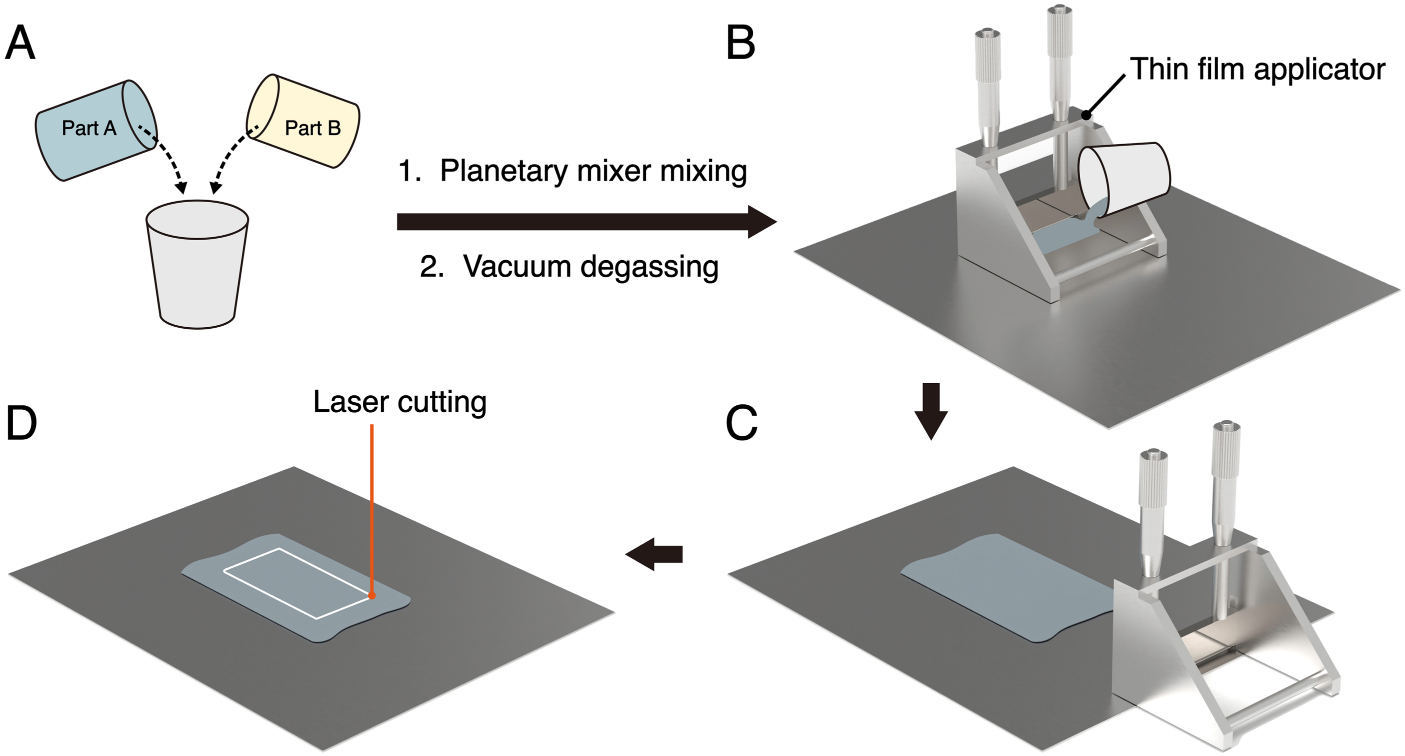

Figure S2: Membrane fabrication process. (*A*) Mixing Part A and Part B of silicone elastomer. (*B*) Pouring the elastomer onto an aluminum sheet and (*C*) adopting the thin film applicator to fabricate a thin layer of elastomer. (*D*) Cutting the membrane into the desired size using CO_2 laser for further prestretch process.

Actuator assembly

After fabricating the frame, SMA coils and wires are inserted into the tunnels of the frame (Fig. S3*A*). It should be noted that the SMA coils inserted are 4 times prestretched from the initial contracted form. Then, two ends of the frame are sealed using silicone elastomer (Dragon-skin 10 NV, Smooth-on, Inc.). Subsequently, the membrane is fixed on a linear stretcher to prestretch to target prestretch ratio λ_p, and two frames are attached to the top and bottom surfaces of the prestretched membrane (Fig. S3*B*). The attaching of the frames can be divided into three major steps: i) The bottom frame is put on a jack platform with a layer of uncured elastomer (Dragon-skin 10 NV, Smooth-on, Inc.) coated to its top surface as the adhesion layer; ii) The jack platform is raised up and the top surface of the bottom frame contacts the bottom of the prestretched membrane. iii) Similarly, the top frame is aligned and positioned on the top surface of the prestretched membrane with a coated adhesion layer on the bottom of the frame. Next, after the adhesion layers are cured and the frames are bonded firmly, the membrane is carefully released from the stretcher and its excessive part will be cut off (Fig. S3*C*), which finally forms the multistable actuator (Fig. S3*D*).

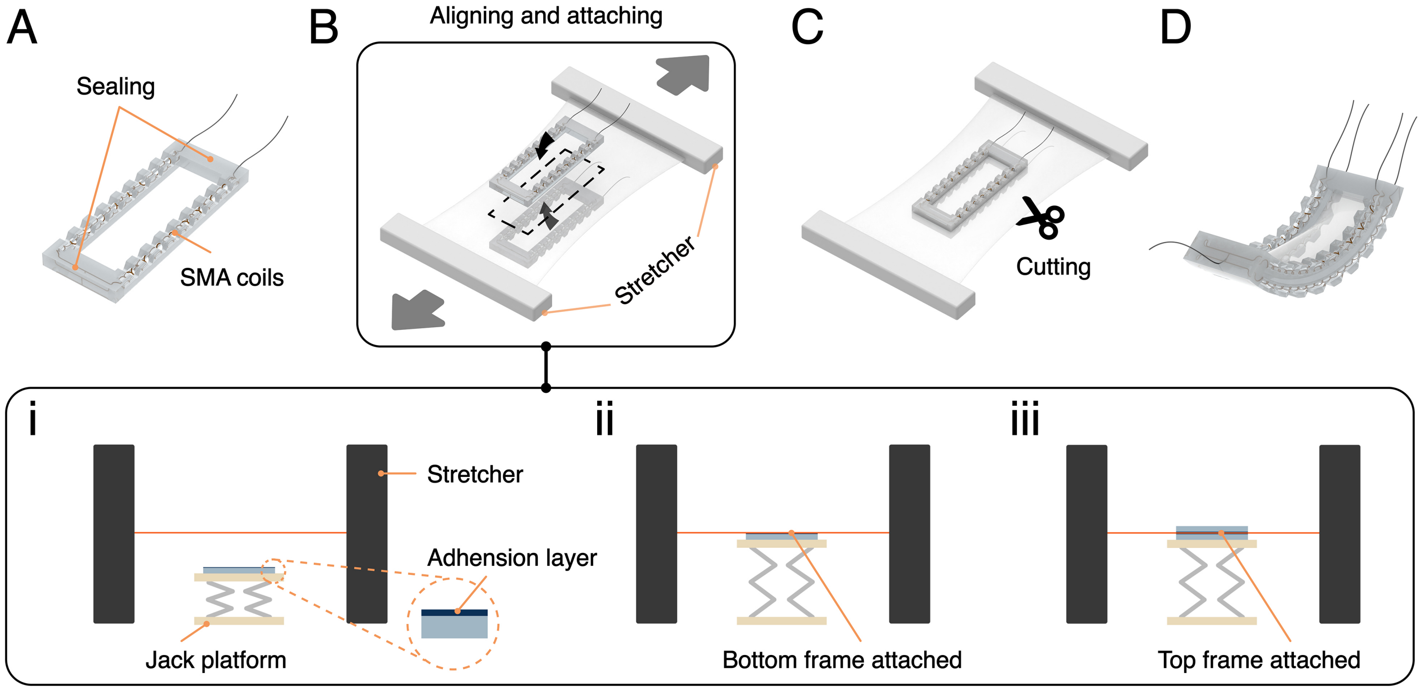

Figure S3: Actuator assembly process. (*A*) Inserting SMA coils into the frame and sealing the two ends of the frame. (*B*) Sandwiching a prestretched membrane with two frames on a stretcher. (*C*) Cutting the excessive part of the membrane. (*D*) Finishing fabrication of the actuator.

Multistable actuator control and SMA nomenclature

We label the SMA coils in the counterclockwise. When the actuator is in state B1, SMA 1 and SMA 4 will be on the side where the actuator is bending towards, and in state B2, SMA 2 and SMA 3 will be on the side where the actuator is bending towards. Fig. S4 illustrates a multistable actuator in stable state B2, with four SMA coils named counterclockwise as SMA1, SMA2, SMA3, and SMA4, respectively. Through the precise control of activation timing for each SMA coil using individual transistors, diverse combinations of SMA activations can be achieved to realize transitions among the six stable states

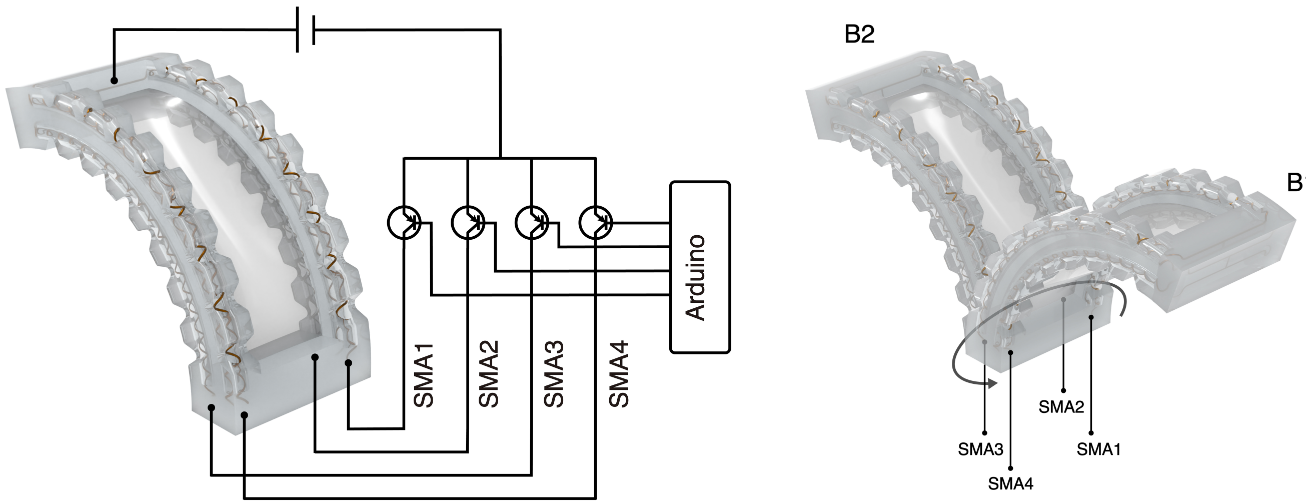

Figure S4: Schematic of the multistable actuator with controls and nomenclature of the distribution and naming of SMA coils.

Actuator Cool down test

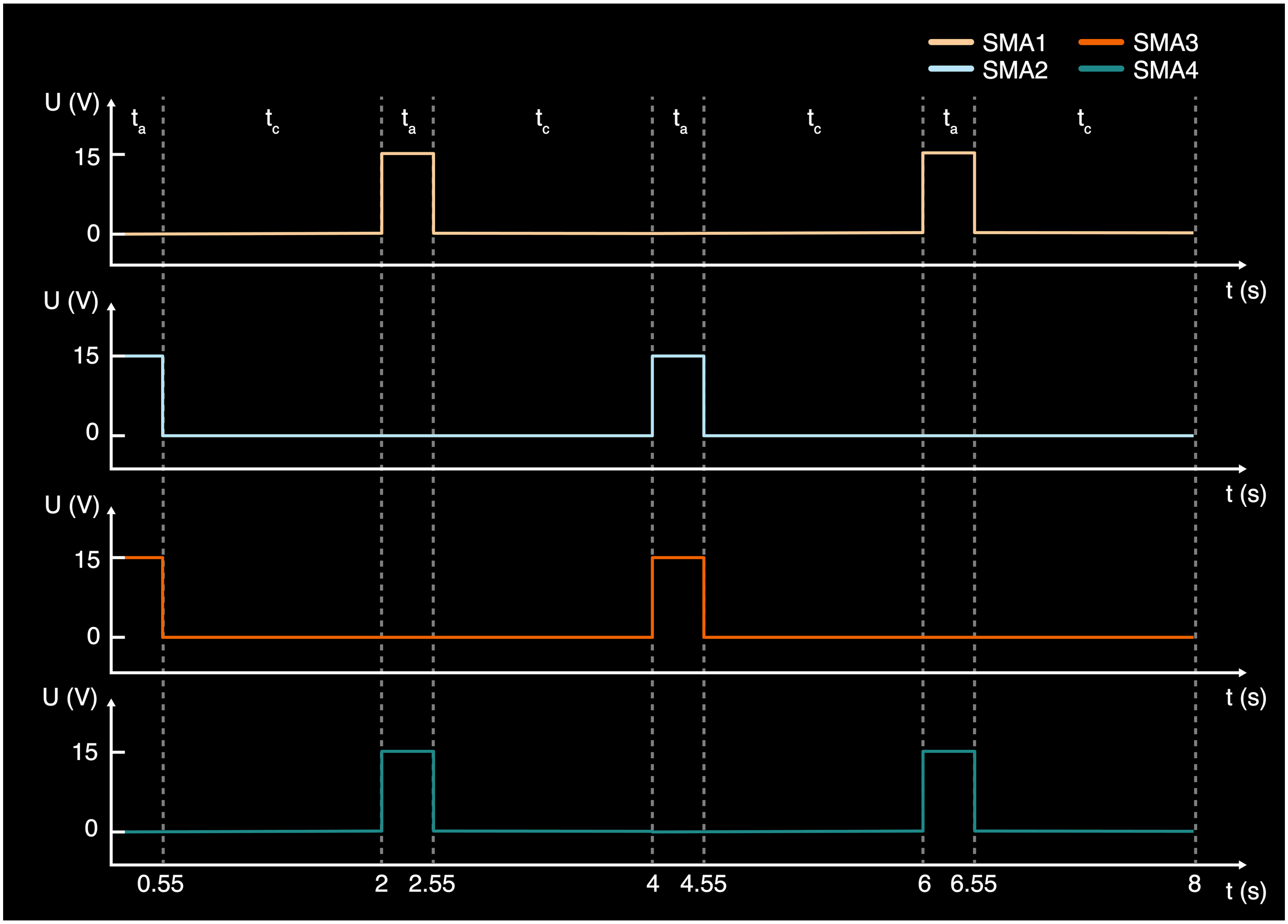

Figure S5: The sequence and duration of SMA activation and cooling at 0.5 Hz.

Actuator overall geometry

As shown in Fig. S6, the actuator's overall geometry is fully defined with two parameters: bending angle θ and twisting angle ϕ. We have the length of the frame's centerline as well as the width of the frame as two constants: L_f, W_f. Using coordinate $O_1 - X_1Y_1Z_1$ as the global coordinate and $O_2 - X_2Y_2Z_2$ as the local coordinate for the tip of the actuator, we have the relationship of the orientation between these two coordinates as following:

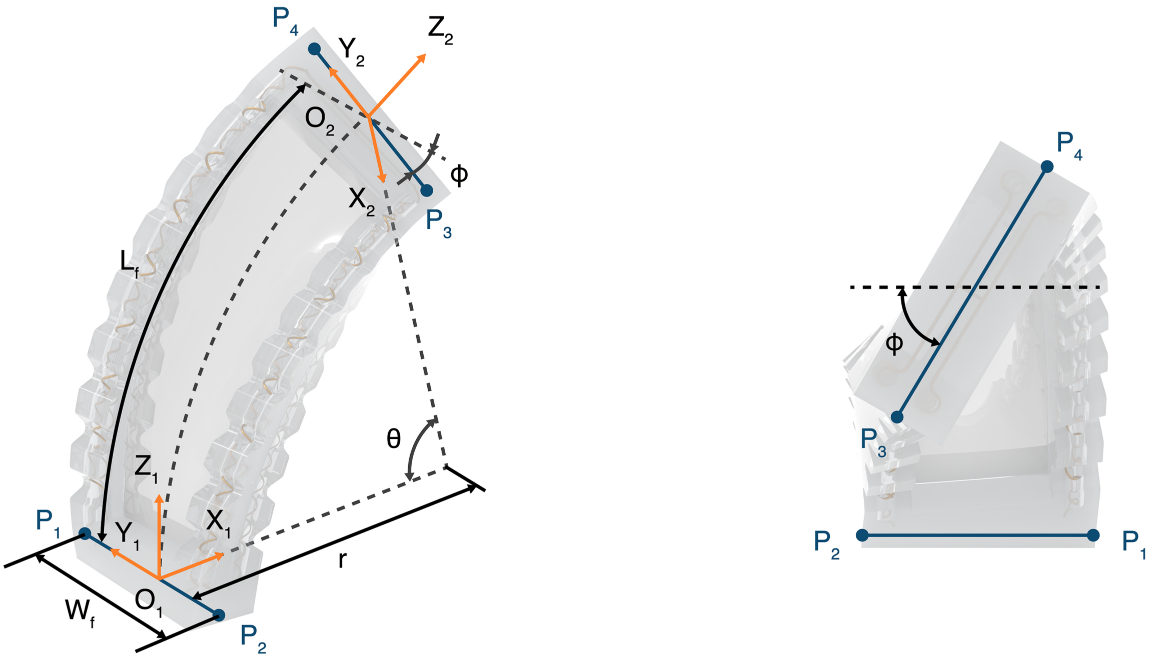

Figure S6: The overall geometry of the actuator

$$\mathbf{R}_{O_1O_2} = \mathbf{R}_y(\theta) \cdot \mathbf{R}_z(\phi) = \begin{bmatrix} cos(\theta)cos(\phi) & -cos(\theta)sin(\phi) & sin(\theta) \\ sin(\phi) & cos(\phi) & 0 \\ -sin(\theta)cos(\phi) & sin(\theta)sin(\phi) & cos(\theta) \end{bmatrix}. \tag{1}$$

The relationship between the bending radius and the bending angle θ is:

$$r = \frac{L_\mathrm{f}}{\theta}, \tag{2}$$

where we can further derive the global position of O_2:

$$\mathbf{O_2} = \begin{bmatrix} r(1 - cos(\theta)) & 0 & rsin(\theta) \end{bmatrix}^\mathrm{T}. \tag{3}$$

Hence, the 4×4 homogeneous transformation matrix between coordinate $O_1 - X_1Y_1Z_1$ and $O_2 - X_2Y_2Z_2$ can be written as:

$$\mathbf{g}_{O_1O_2} = \begin{bmatrix} & \mathbf{R}_{O_1O_2} & & \mathbf{O_2} \\ 0 & 0 & 0 & 1 \end{bmatrix}. \tag{4}$$

The global position of the bottom two edges of the frame in homogeneous form can be directly written as:

$$\mathbf{P}_1 = \begin{bmatrix} 0 & \frac{W_f}{2} & 0 & 1 \end{bmatrix}^\mathrm{T}, \tag{5}$$

$$\mathbf{P}_2 = \begin{bmatrix} 0 & -\frac{W_f}{2} & 0 & 1 \end{bmatrix}^\mathrm{T}. \tag{6}$$

while the local position (in coordinate $O_2 - X_2Y_2Z_2$) of the top two edges of the frame in the homogeneous form are:

$$\mathbf{P}_{3_{O_2}} = \begin{bmatrix} 0 & -\frac{W_f}{2} & 0 & 1 \end{bmatrix}^\mathrm{T}, \tag{7}$$

$$\mathbf{P}_{4_{O_2}} = \begin{bmatrix} 0 & \frac{W_f}{2} & 0 & 1 \end{bmatrix}^{\mathrm{T}} . \tag{8}$$

Therefore, we have their global positions as:

$$\mathbf{P}_3 = \mathbf{g}_{O_1 O_2} \cdot \mathbf{P}_{3_{O_2}} , \tag{9}$$

$$\mathbf{P}_4 = \mathbf{g}_{O_1 O_2} \cdot \mathbf{P}_{4_{O_2}} . \tag{10}$$

The above-mentioned equations provide us with a full description of the overall parameterized geometry of the actuator. While further description of the detailed geometry could be found in Section. .

Motion capture process

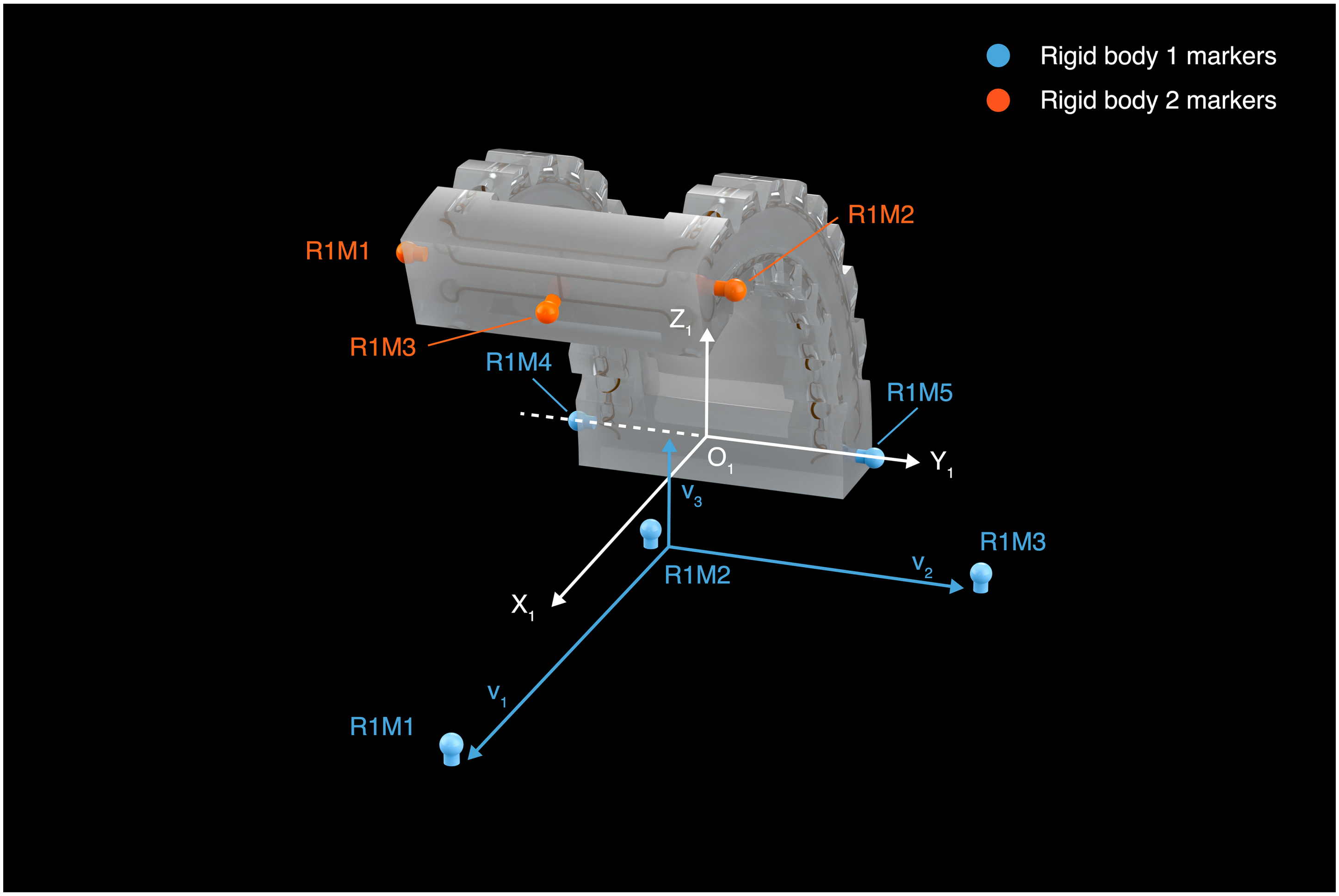

Figure S7: Distribution of markers

The purpose of the mocap system (Motive, OptiTrack) is to use captured positions of the markers to estimate the closest configuration parameter [θ, ϕ]. As depicted in Fig. S7, in total eight markers (6.4mm M3 Markers, MKR064M3-10, OptiTrack) are adopted, five installed on the actuator and three installed on the platform. Since the actuator is highly dynamic, it is important to form rigid bodies locally so that even when the system lost the information of one of the markers, it can still generate it based on the other markers within the local rigid body. Hence, three platform markers together with the bottom two actuator markers form rigid body No.1 while the top three markers on the tip of the actuator form rigid body No.2.

Rigid body No.1 is steady during the transition. R1M1, R1M2, and R1M3 establish the normal of the platform:

$$\mathbf{v}_3 = \mathbf{v}_1 \times \mathbf{v}_2 , \tag{11}$$

which further forms the Z_1 axis of coordinate $O_1 - X_1Y_1Z_1$. Next, the vector pointing from R1M4 to R1M5 forms the Y_1 axis of coordinate $O_1 - X_1Y_1Z_1$. Thereby, the global coordinate $O_1 - X_1Y_1Z_1$ has been fully established.

Subsequently, the three markers in rigid body No.2, R2M1, R2M2, and R2M3, are converted into the form expressed under the global coordinate $O_1 - X_1Y_1Z_1$, and then fitting the closest configuration parameter [θ, ϕ] using Fminsearch in MATLAB. Consequently, we are able to convert the highly dynamic mocap information into actuator configuration information.

We also present the angular data collected via the mocap system during the experiment. As illustrated in Fig. S8, the actuator repeats the transition between stable states B1 and B2 back and forth 14 times (i.e. 7 cycles).

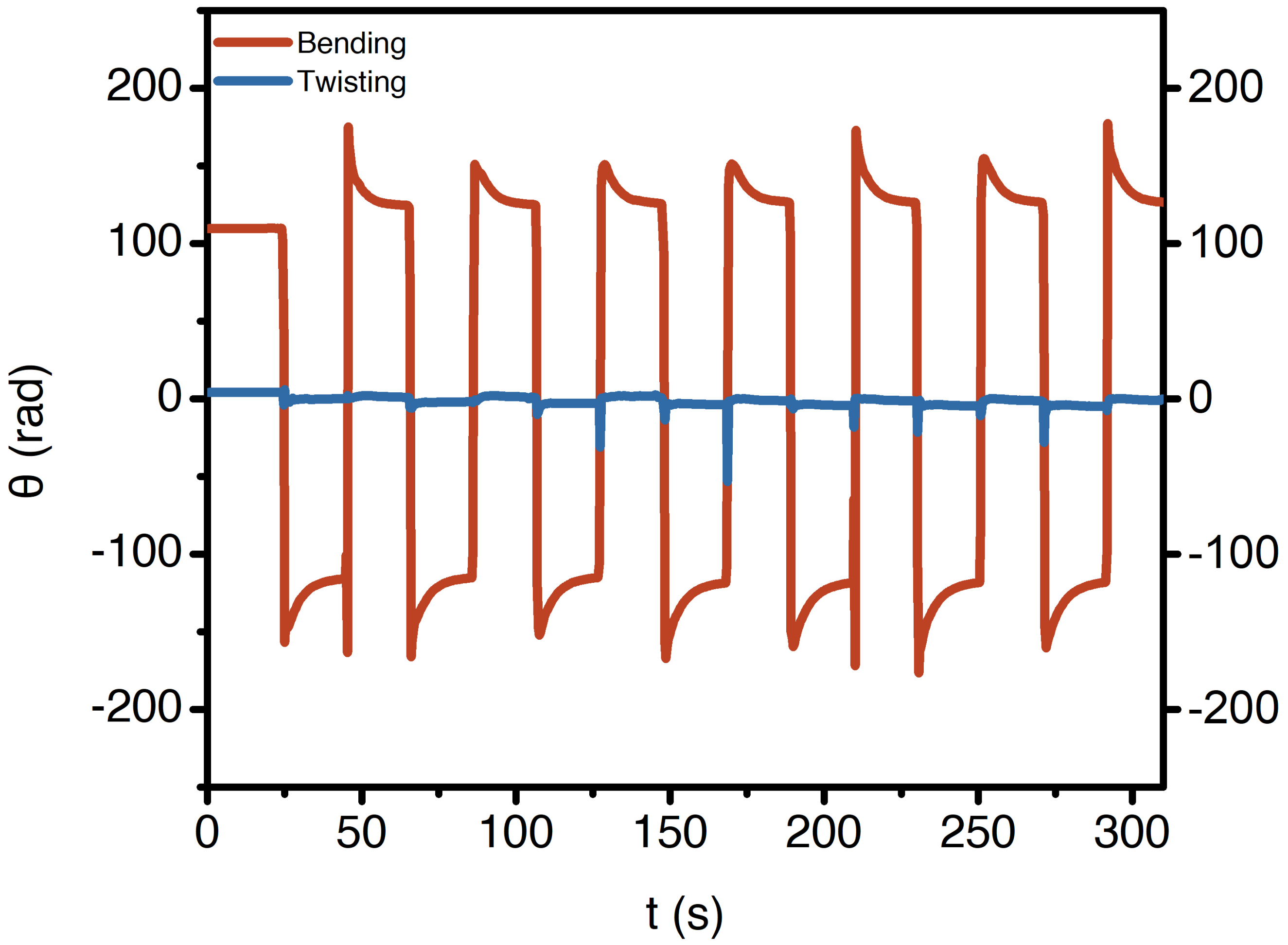

Figure S8: Angular data of repeating transition between two bending stable states

Analytic model

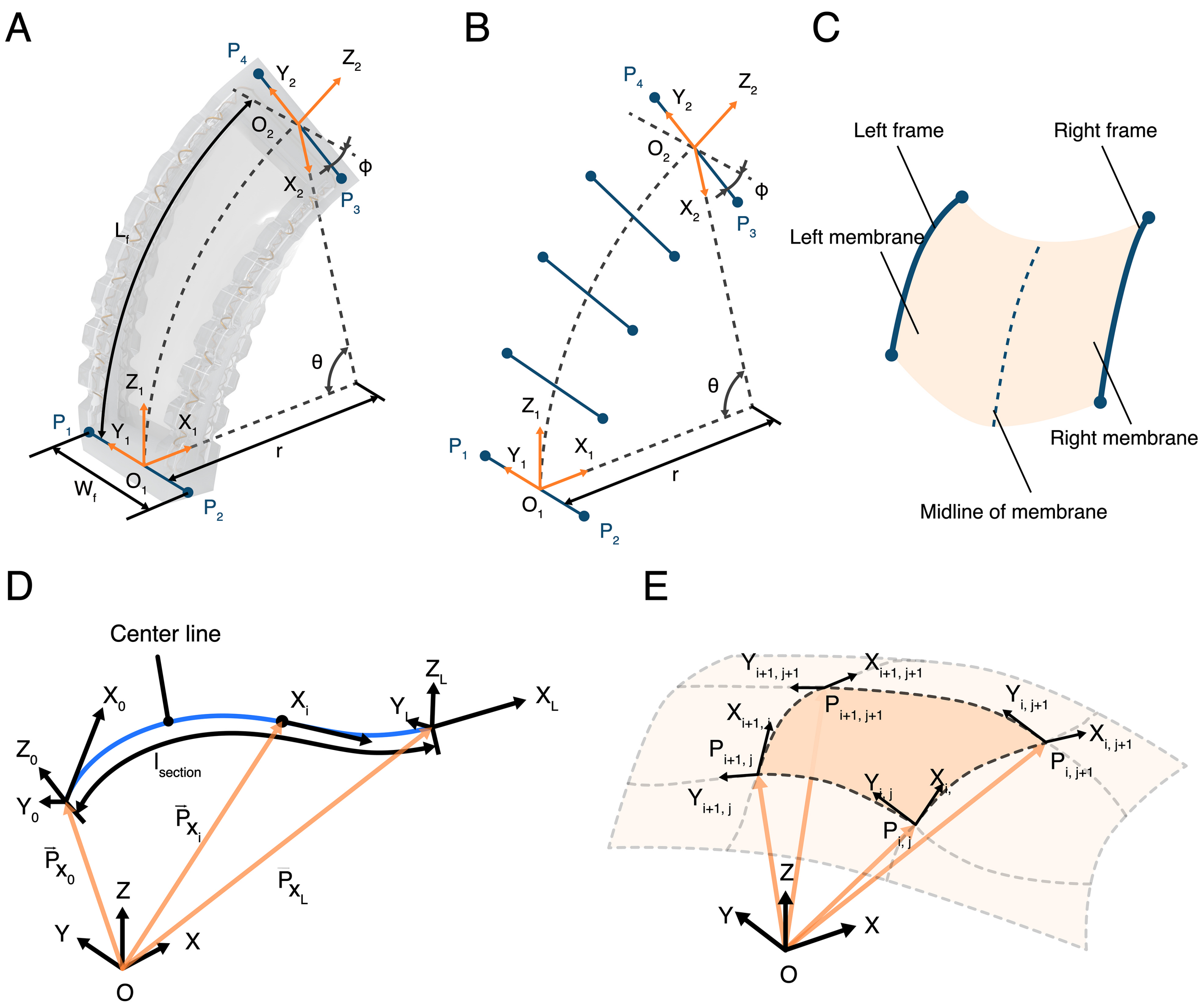

Figure S9: The analytic model

Based on our previous estimation of the geometry (Fig. S9*A*), we can further estimate a uniform bending and twisting on the virtual centerline between O_1 and O_2. Hence, we can divide the frame into several sections uniformly. For each section, Absolute Nodal Coordinate Formulation (ANCF) is adopted to calculate the elastic potential energy [1, 2, 3, 4, 5]. The reason for dividing the actuator into sections is that when the curvature is relatively large, the ANCF method might deviate from the actual geometry, which results in errors. In practice, we divide the frame and the membrane into 10 sections.

As for each section, it is considered to be a left frame and a right frame with a membrane in the middle. The membrane is further divided into the left parts and the right parts.

For the frame part, we adopt beam equations to describe its geometry:

$$\mathbf{P}_{\text{frame}}(X,Y,Z)_{3\times1} = \mathbf{S}_{\text{frame}}(X,Y,Z)_{3\times24} \cdot \mathbf{q}^e{}_{24\times1}\,, \tag{12}$$

where $\mathbf{S}_{\text{frame}}$ is the shape matrix and $\mathbf{q}^e$ is the boundary condition of this frame section:

$$\mathbf{S}_{frame}(X,Y,Z)_{3\times24} = \begin{bmatrix} s_1\mathbf{I} & s_2\mathbf{I} & s_3\mathbf{I} & s_4\mathbf{I} & s_5\mathbf{I} & s_6\mathbf{I} & s_7\mathbf{I} & s_8\mathbf{I} \end{bmatrix}, \tag{13}$$

$$\mathbf{q}^e{}_{24\times1} = \begin{bmatrix} \mathbf{P}_0{}^{\mathrm{T}} & \frac{\partial \mathbf{P}_0}{\partial X_0}^{\mathrm{T}} & \frac{\partial \mathbf{P}_0}{\partial Y_0}^{\mathrm{T}} & \frac{\partial \mathbf{P}_0}{\partial Z_0}^{\mathrm{T}} & \mathbf{P}_L{}^{\mathrm{T}} & \frac{\partial \mathbf{P}_L}{\partial X_L}^{\mathrm{T}} & \frac{\partial \mathbf{P}_L}{\partial Y_L}^{\mathrm{T}} & \frac{\partial \mathbf{P}_L}{\partial Z_L}^{\mathrm{T}} \end{bmatrix}^{\mathrm{T}}. \tag{14}$$

The parameters in the shape matrix are calculated as the following:

$$s_1 = 1 - \frac{3X^2}{{l_f}^2} + \frac{2X^3}{{l_f}^3}\,. \tag{15}$$

$$s_2 = X - \frac{2X^2}{l_f} + \frac{X^3}{{l_f}^2}\,. \tag{16}$$

$$s_3 = Y - \frac{XY}{l_f}\,. \tag{17}$$

$$s_4 = Z - \frac{XZ}{l_f}\,. \tag{18}$$

$$s_5 = \frac{3X^2}{{l_f}^2} - \frac{2X^3}{{l_f}^3}\,. \tag{19}$$

$$s_6 = -\frac{X^2}{l_f} + \frac{X^3}{{l_f}^2}\,. \tag{20}$$

$$s_7 = \frac{XY}{l_f}\,. \tag{21}$$

$$s_8 = \frac{XZ}{l_f}\,. \tag{22}$$

$$s_8 = \frac{XZ}{l_f}\,. \tag{23}$$

where l_f is the length of this frame section. Next, we calculate the curvature of the frame's centerline as follows:

$$\mathbf{p_y}(X) = \left.\frac{\partial \mathbf{P}(X,Y,Z)}{\partial Y}\right|_{Y,Z=0} \tag{24}$$

$$\mathbf{p_z}(X) = \left.\frac{\partial \mathbf{P}(X,Y,Z)}{\partial Z}\right|_{Y,Z=0} \tag{25}$$

$$\mathbf{R}(X) = \begin{bmatrix}\mathbf{p_y}\times\mathbf{p_z} & \mathbf{p_y} & \mathbf{p_z}\end{bmatrix}_{3\times3} \tag{26}$$

$$\begin{bmatrix} 0 & -\kappa_y(X) & \kappa_z(X) \\ \kappa_y(X) & 0 & -\kappa_t(X) \\ -\kappa_z(X) & \kappa_t(X) & 0 \end{bmatrix}_{3\times3} = \mathbf{R}(X)^{\mathrm{T}}\frac{\partial \mathbf{R}(X)}{\partial X} \tag{27}$$

The frame's two-way bending stiffness and the torsional stiffness are denoted as D_y, D_z, and D_t. Hence, the elastic potential energy of one side of the frame is derived as:

$$U_{\text{frame}} = \frac{1}{2}\int_0^{l_f}(\kappa_y D_y \kappa_y + \kappa_z D_z \kappa_z + \kappa_t D_t \kappa_t)dX\,. \tag{28}$$

By summing up the energy of the left and right parts, we have the total potential energy of the entire frame.

For the membrane part, since one side of the membrane is attached to the frame, we can assume it shares the same boundary conditions as the frame on this side. On the other side, which is the midline of the membrane, we introduce an optimizable parameter κ_{mid} to describe the geometry. Denote the bending curvature of the virtual midline of the frame as κ_{max}. It is obvious that $0 \leq \kappa_{mid} \leq \kappa_{max}$.

By giving this κ_{mid}, we can assume the entire shape of the midline of the membrane using the same ANCF beam equation. Then, for each section of the membrane, we have the boundary condition of its four edges and further apply the ANCF membrane equation to it:

$$\mathbf{P}_{\text{membrane}}(X,Y)_{3\times1} = \mathbf{S}_{\text{membrane}}(X,Y)_{3\times36}\cdot {\mathbf{q}^e}_{36\times1}\,. \tag{29}$$

$$\mathbf{S}_{\text{membrane}}(X,Y)_{3\times36} = \begin{bmatrix} s_1\mathbf{I} & s_2\mathbf{I} & s_3\mathbf{I} & s_4\mathbf{I} & s_5\mathbf{I} & s_6\mathbf{I} & \\ & s_7\mathbf{I} & s_8\mathbf{I} & s_9\mathbf{I} & s_{10}\mathbf{I} & s_{11}\mathbf{I} & s_{12}\mathbf{I} \end{bmatrix} \tag{30}$$

$$\mathbf{q}_{\mathrm{i,j}\,9\times1} = \begin{bmatrix}{\mathbf{P}_{\mathrm{i,j}}}^{\mathrm{T}} & \frac{\partial \mathbf{P}\mathrm{i,j}}{\partial X_{\mathrm{i,j}}}^{\mathrm{T}} & \frac{\partial \mathbf{P}_{\mathrm{i,j}}}{\partial Y_{\mathrm{i,j}}}^{\mathrm{T}}\end{bmatrix}^{\mathrm{T}} \tag{31}$$

$$\mathbf{q}^{e}{}_{36\times1} = \left[\mathbf{q}_{\mathrm{i,j}}{}^{\mathrm{T}} \quad \mathbf{q}_{\mathrm{i,j+1}}{}^{\mathrm{T}} \quad \mathbf{q}_{\mathrm{i+1,j+1}}{}^{\mathrm{T}} \quad \mathbf{q}_{\mathrm{i+1,j}}{}^{\mathrm{T}}\right]^{\mathrm{T}} \tag{32}$$

$\mathbf{q}_{i,j}$ is the boundary condition derived from the beam equation of the frame. It should be noted that in order to consider the prestretch, a constant λ_p should be multiplied on the derivative on the X axis of the membrane.

As for the parameters within the shape matrix of the membrane, we have:

$$s_1 = \frac{2Y^3}{W^3} - \frac{3X^2}{W^2} - \frac{3Y^2}{L^2} + \frac{2X^3}{L^3} + \frac{3XY^2}{LW^2} + \frac{3X^2Y}{L^2W} - \frac{2XY^3}{LW^3} - \frac{2X^3Y}{L^3W} - \frac{XY}{LW} + 1 \tag{33}$$

$$s_2 = X - \frac{2X^2}{L} + \frac{X^3}{L^2} - \frac{XY}{W} + \frac{2X^2Y}{LW} - \frac{X^3Y}{L^2W} \tag{34}$$

$$s_3 = Y - \frac{2Y^2}{W} + \frac{Y^3}{W^2} - \frac{XY}{L} + \frac{2XY^2}{LW} - \frac{XY^3}{LW^2} \tag{35}$$

$$s_4 = \frac{3X^2}{L^2} - \frac{2X^3}{L^3} - \frac{3XY^2}{LW^2} - \frac{3X^2Y}{L^2W} + \frac{2XY^3}{LW^3} + \frac{2X^3Y}{L^3W} + \frac{XY}{LW} \tag{36}$$

$$s_5 = \frac{X^3}{L^2} - \frac{X^2}{L} + \frac{X^2Y}{LW} - \frac{X^3Y}{L^2W} \tag{37}$$

$$s_6 = \frac{XY}{L} - \frac{2XY^2}{LW} + \frac{XY^3}{LW^2} \tag{38}$$

$$s_7 = \frac{3XY^2}{LW^2} + \frac{3X^2Y}{L^2W} - \frac{2XY^3}{LW^3} - \frac{2X^3Y}{L^3W} - \frac{XY}{LW} \tag{39}$$

$$s_8 = \frac{X^3Y}{L^2W} - \frac{X^2Y}{LW} \tag{40}$$

$$s_9 = \frac{XY^3}{LW^2} - \frac{XY^2}{LW} \tag{41}$$

$$s_{10} = \frac{3Y^2}{W^2} - \frac{2Y^3}{W^3} - \frac{3XY^2}{LW^2} - \frac{3X^2Y}{L^2W} + \frac{2XY^3}{LW^3} + \frac{2X^3Y}{L^3W} + \frac{XY}{LW} \tag{42}$$

$$s_{11} = \frac{XY}{W} - \frac{2X^2Y}{LW} + \frac{X^3Y}{L^2W} \tag{43}$$

$$s_{12} = \frac{Y^3}{W^2} - \frac{Y^2}{W} + \frac{XY^2}{LW} - \frac{XY^3}{LW^2} \tag{44}$$

By taking the derivative in respect of X and Y, we have:

$$r_X = \frac{\partial \mathbf{P}_{membrane}(X,Y)}{\partial X} \tag{45}$$

$$r_Y = \frac{\partial \mathbf{P}_{membrane}(X,Y)}{\partial Y} \tag{46}$$

Since we assume the elastomer to be incompressible, we have:

$$I_1 = r_X^T \cdot r_X + r_Y^T \cdot r_Y + \frac{1}{(r_X^T \cdot r_X)\cdot(r_Y^T \cdot r_Y)}\,, \tag{47}$$

And further, the strain energy density using Yeoh hyperelastic model[6] is obtained as:

$$W_{\mathrm{Yeoh}} = c_1 \cdot (I_1 - 3) + c_2 \cdot (I_1 - 3)^2 + c_3 \cdot (I_1 - 3)^3\,. \tag{48}$$

Subsequently, the total elastic potential energy of one side of the membrane is derived as:

$$U_{\mathrm{membrane}} = H \cdot \int_0^W \int_0^L W_{\mathrm{Yeoh}}\, dX dY\,, \tag{49}$$

where L, W, and H are the initial length, width, and thickness of the elastic membrane.

The elastic coefficients needed to establish the hyperelastic (constitutive) behavior are obtained by conducting an experimental tensile test (Instron 5969, Universal Testing Machine equipped with 50 N load cell) on the material. The sample is laser-cut into the shape of ASTM D412 Type Die-C dogbone coupons and the longest dimension is placed in the tensile direction. The strain rate for this test is 10 mm/min. Based on the recorded stretch-stress data, curve-fitting is adopted to get hyperelastic model parameters. The result is shown in Fig. S10. The fitted parameters are: C_1 = 82752.5936 Pa, C_2 = 10215.42628 Pa, C_3 = -438.9334941 Pa. The bending stiffness and the torsional stiffness of the frame are tested by hanging weights to the tip of the frame. The results are: $D_y = 2.102457 \times 10^{-4}$ N·m^2, $D_z = 1.13775 \times 10^{-4}$ N·m^2, and $D_t = 2.91206 \times 10^{-4}$ N·m^2.

Finally, we test our model on a specific prestretch ratio $\lambda_p = 2$, where the model predicts two pure-bend states on $\theta = \pm$ 1.65 rad, as shown in Fig. S11. Which is close but slightly smaller than the experimental data. This could be explained by the effect of SMA coils. Although SMA coils do not affect the development of multistability, they do affect the final value of the convergence since they are pseudo-elastic.

We also developed a simplified analytic model to study pure-bend stable states. In this case, the geometric parameter is reduced to one: bending angle θ. In order to simplify the geometry of the elastic membrane, we also assume it is only connected to the two extremities of the bending frame and treated as a two-dimensional thin film. Therefore, the local stretch ratio on the membrane could be uniformly expressed as:

$$\lambda_1(\theta, \lambda_p) = \frac{2\lambda_p \sin\frac{\theta}{2}}{\theta}\,. \tag{50}$$

Therefore, we can derive the elastic potential energy of the simplified membrane and further derive the total elastic potential energy of the actuator as:

$$U_{\text{total}}(\theta, \lambda_p) = U_{\text{mem}}(\theta, \lambda_p) + U_{\text{frame}}(\theta)\,. \tag{51}$$

By taking the derivative of the total elastic potential energy of the actuator to θ:

$$\frac{\partial U_{\text{total}}}{\partial \theta} = 0\,. \tag{52}$$

we can predict the stable state angle of the pure-bending state as a function of the prestretch ratio λ_p using this simplified analytic model. The result of this simplified model as well as the comparison to the experimental data is plotted in Fig. S12.

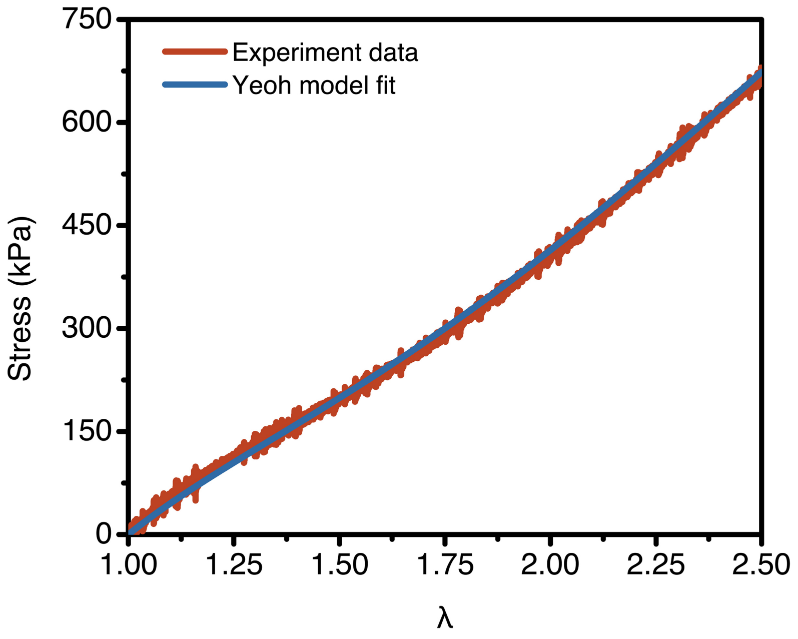

Figure S10: Fitting hyperelastic parameters

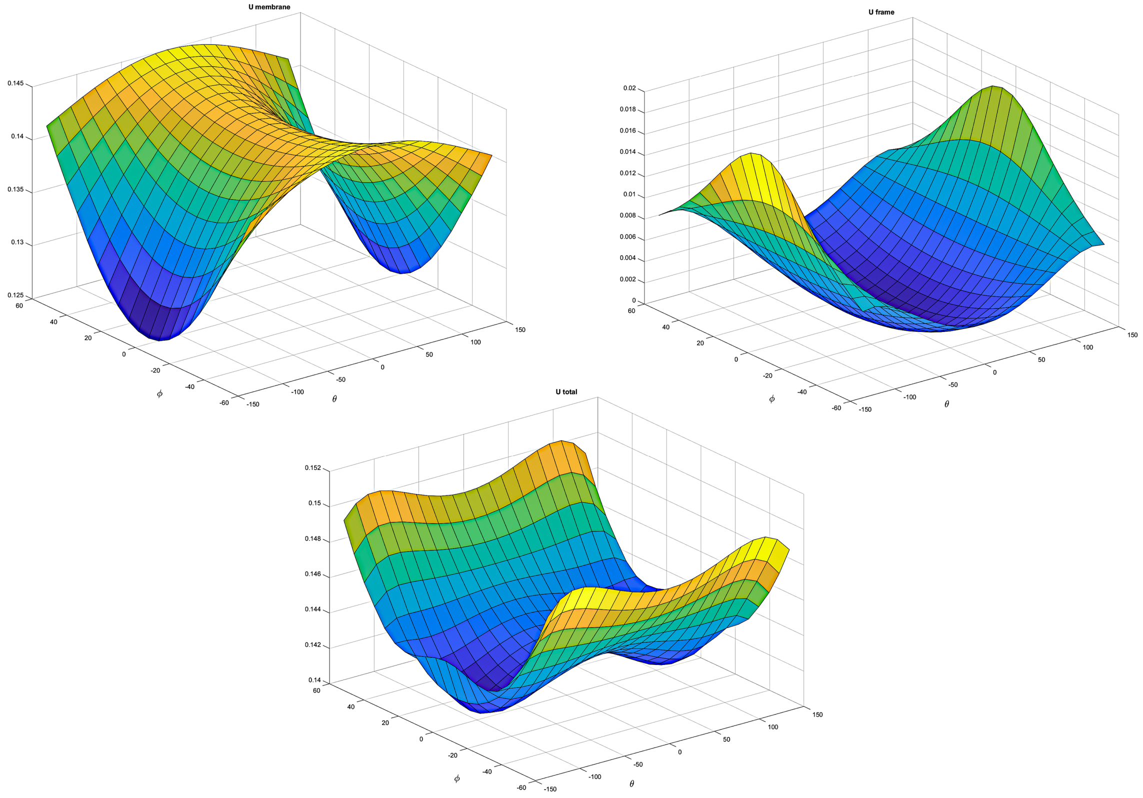

Figure S11: The result on elastic potential energy

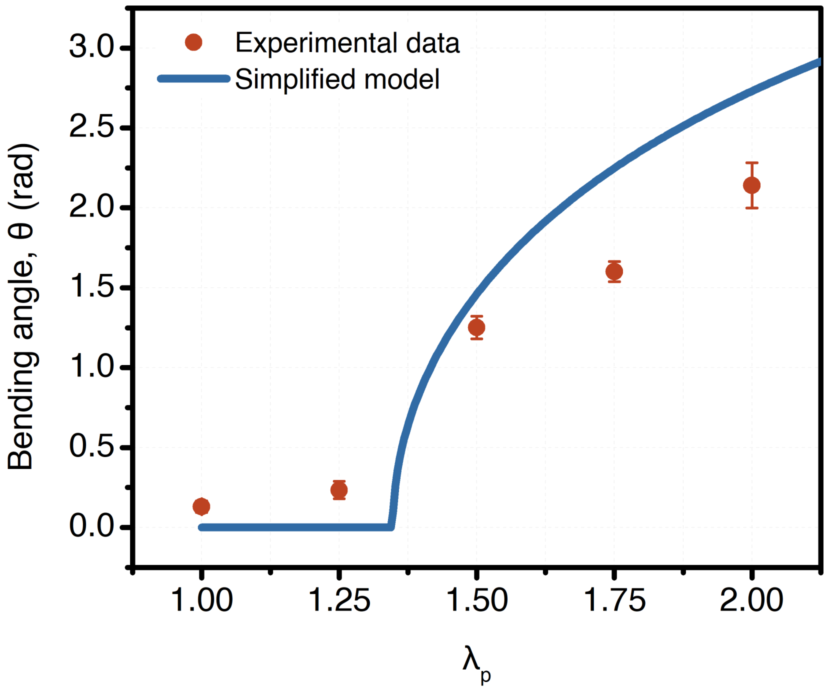

Figure S12: The simplified model's prediction of the stable-state angle compared to the experimental data points for pure-bend stable states.

Heliotropism inspired energy harvesting

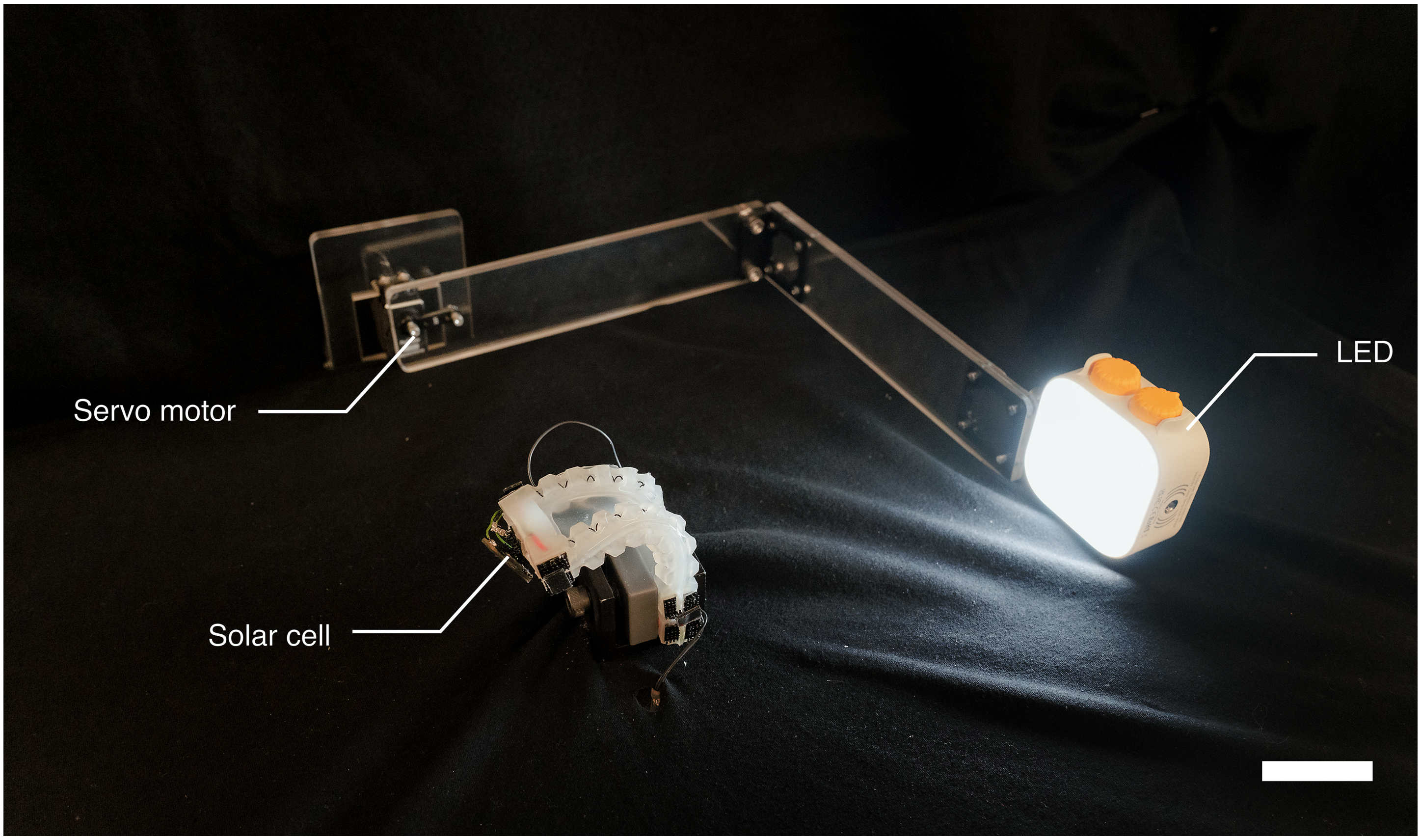

Figure S13: Heliotropism inspired energy harvesting experiment setup. Scale bar: 40 mm.

The experiment setup is shown in Fig. S13. An LED (Tofu RGB Video Light, PHOTOOLEX) is installed on an acrylic limb driven by a servo motor (579MG, Power Hobbies) causing it to move in a circular path with a cycle time of t = 90 s to mimic the sun's movement during the daytime. A miniature photovoltaic cell (KXOB25-14X1F-TB, ANYSOLAR Ltd.) is mounted on the tip of the actuator for energy harvesting. The photovoltaic cell is serially connected to a 10 Ω resistor while a multimeter is paralleled connected to the resistor to measure the applied voltage. Hence, we can estimate the output power of the photovoltaic cell as:

$$\mathbf{P}_{\text{output}} = \frac{U^2}{R}. \tag{53}$$

It should be noted that the purpose of this demonstration is a 'proof-of-concept' that by introducing multiple stable states, the actuator is capable of tracking the movement of the light source to achieve better energy output without continuous energy input. During the 90 s experiment, the energy output from the miniature photovoltaic cell at each single stable state are 2.47×10^{-5} J (B1), 7.38×10^{-5} J (T1$^+$), 4.35×10^{-5} J (T2$^+$), and 5.92×10^{-5} J (B2), respectively. By involving multiple stable states, the energy output of the miniature photovoltaic cell is improved to 2.02×10^{-4} J. By comparing the energy output of a single stable state and multiple states, we observe the output energy improved by about one order of magnitude.

During the experiment, the input energy for the actuator is 35.14 ± 0.13 J for 550 ms of activation to transition from one pure-bend state to the opposite pure-bend state. This disproportionality between the consumed energy and generated energy is primarily because: 1) The light source involved in this experiment is a mini-LED panel with a maximum illumination of about 900 lux. On the contrary, the sun's illumination is at the magnitude of several tens of thousands of lux to hundreds of thousands of lux. 2) The photovoltaic cell employed in this experiment is a miniatured one with limitation on energy output (P_{max} = 30.7 mW). 3) The actuation mechanism for our multistable actuator is the SMA coil, which is rather power-hungry and low-efficient. The energy efficiency of this demonstration could be potentially improved by triggering the actuator via other actuation mechanisms with higher energy efficiency, employing a better miniature photovoltaic cell with a higher output power limit, and using a stronger light source to better mimic the sun's illumination.

Dexterously-turning crawler

A dexterously-turning crawler is fabricated based on a multistable actuator and two pairs of directionally asymmetric friction feet. By controlling the activation and cooling sequence and duration of SMA coils, the crawler can utilize its multiple stable states to crawl forward and turn directions dexterously. The detailed plot for SMA activation and cooling for crawling, turning right, and turning left are illustrated in Fig S14, S15, and S16, respectively.

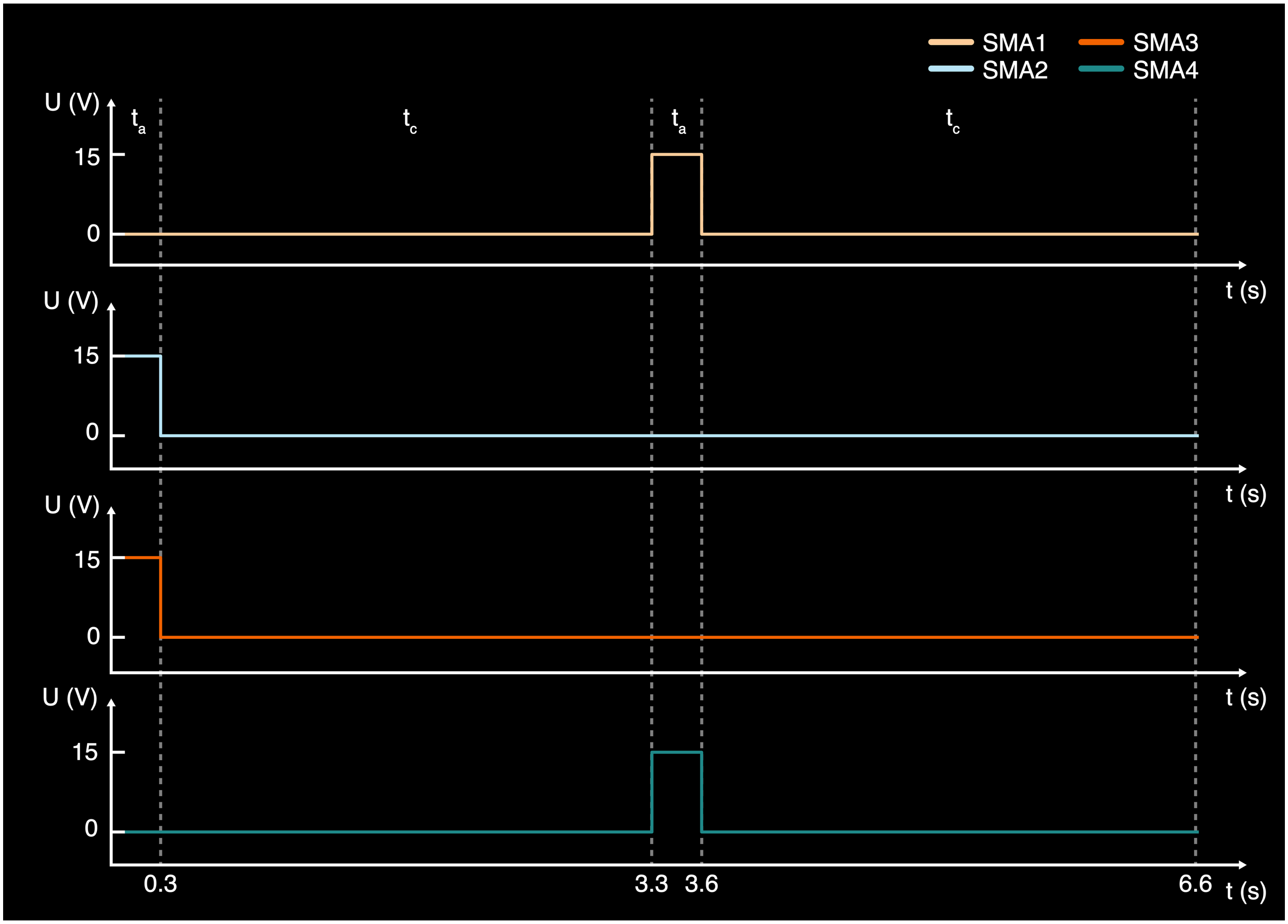

Figure S14: The sequence and duration of SMA activation and cooling for crawling.

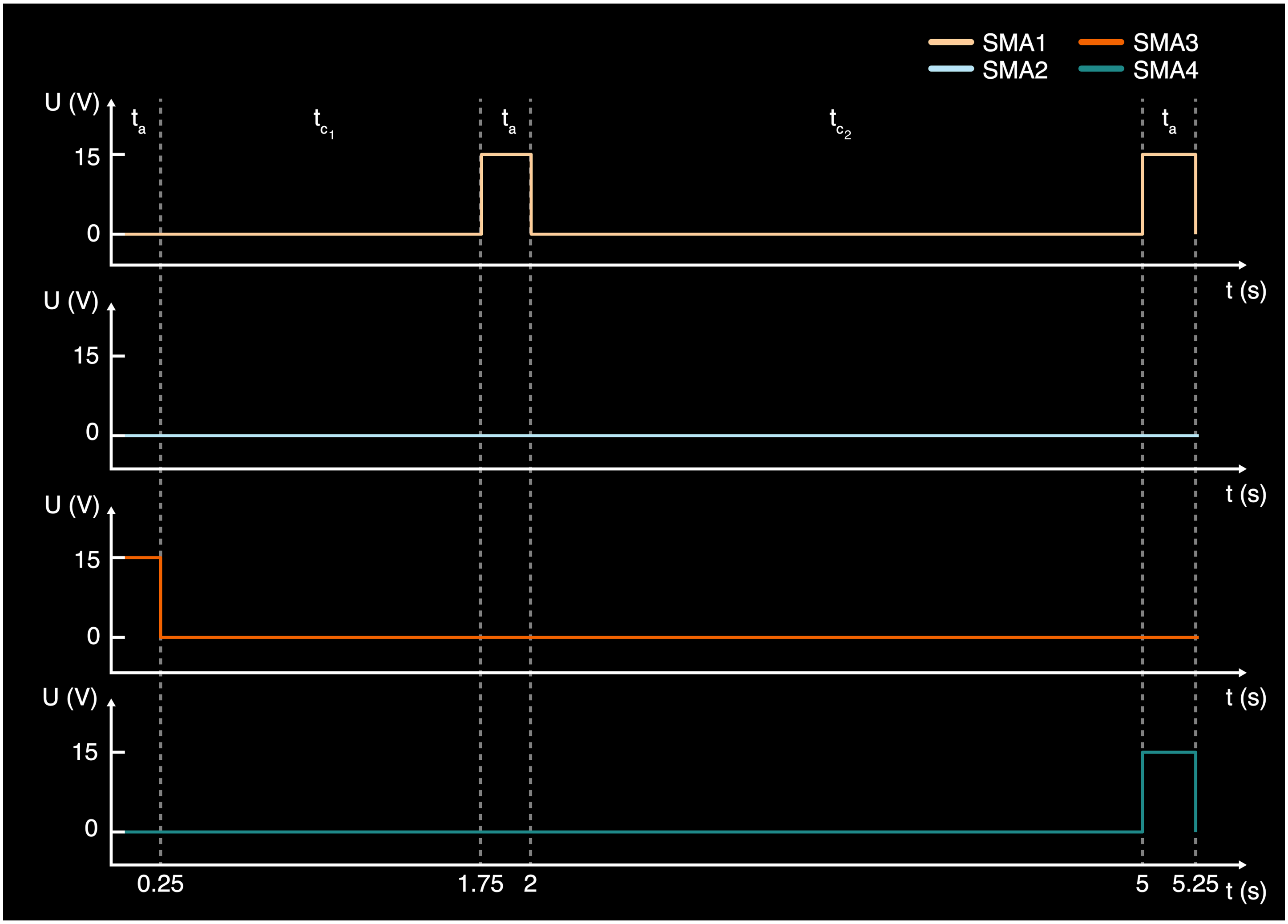

Figure S15: The sequence and duration of SMA activation and cooling for turning right.

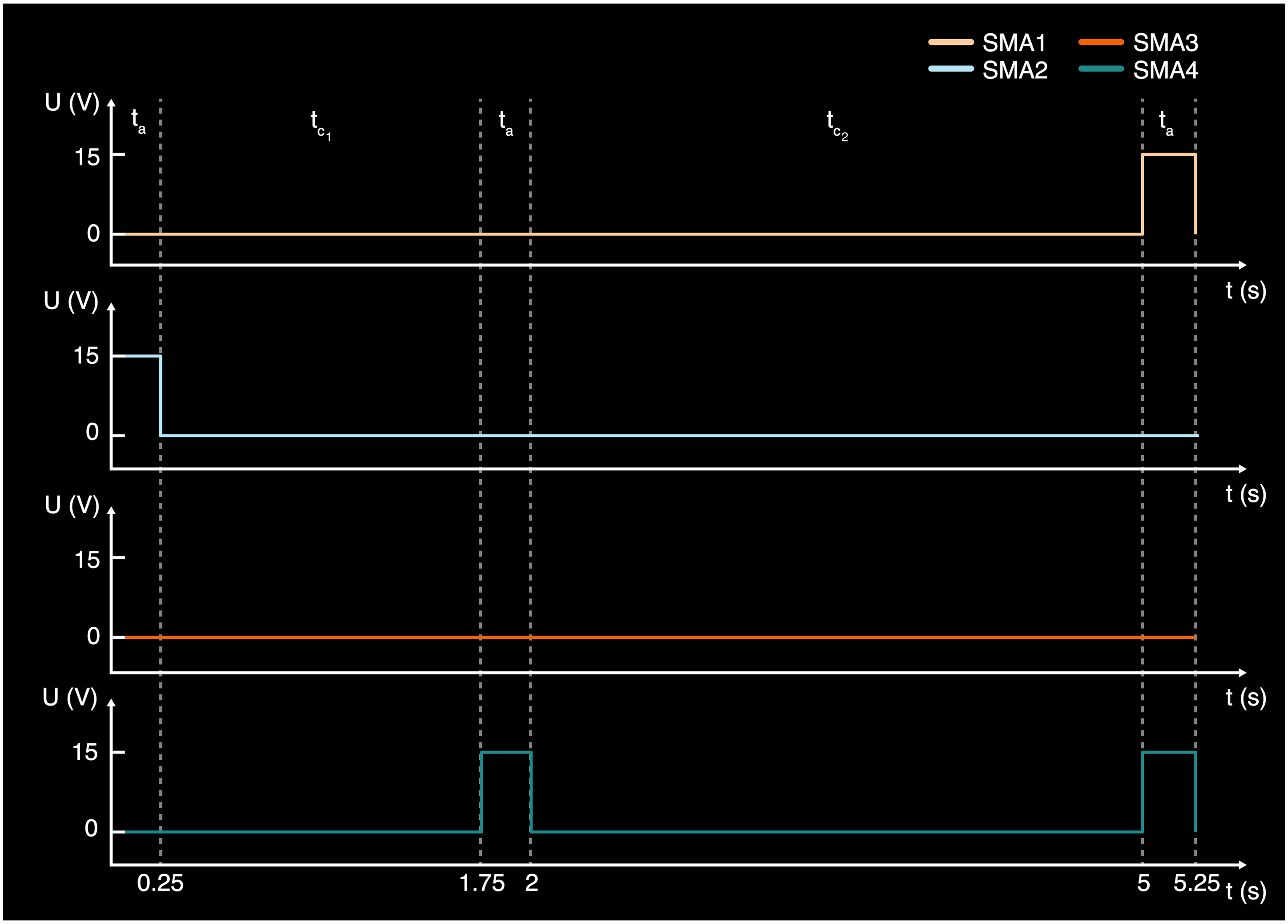

Figure S16: The sequence and duration of SMA activation and cooling for turning left.

A passive multistable structure

We replace SMA coils with 3D-printed elastic rods (Flexible 80A, Formlabs Inc.) to construct a fully passive multistable structure. Such a passive structure also shows six stable states (Fig. S17). The manual transition of this passive structure is shown in Movie S7.

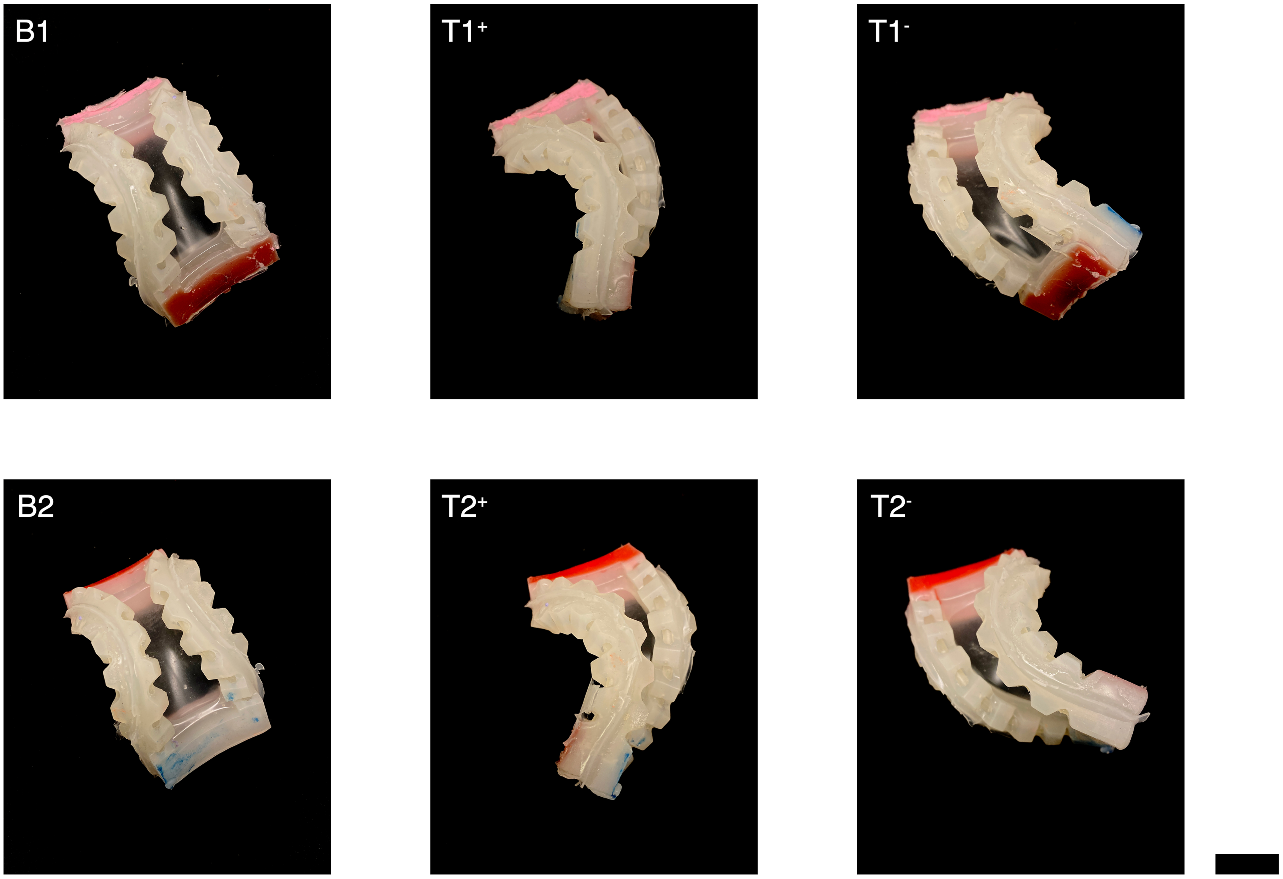

Figure S17: A passive multistable structure. Scale bar: 20 mm.

Comparison of various snap-through soft actuators

Table S1: **Snap-through soft actuator comparison.**

**Pre-load element**	**Constraint element**	**Actuation mechanism**	**Actuation frequency (Hz)**	**Multistability**	**Number of units**	**Referrence**
Ferroelastomer	Rigid support	Magnetic	10	No	1	[7]
Ferroelastomer	Rigid support	Magnetic	~ 5	No	1	[8]
Origami paper	Origami paper	Magnetic	$0.3 \sim 5$	Yes	≥ 2	[9]
Origami paper	Origami paper	Magnetic	~ 0.33	Yes	≥ 2	[10]
Liquid crystal elastomer	Plastic frame	Light	$0.5 \sim 3$	No	1	[11]
Dielectric elastomer	PET support layer	DEA	~ 0.1	No	1	[12]
Dielectric elastomer	Acrylic frame	DEA	~ 0.02	Yes	$2 \sim 5$	[13]
Silicone elastomer	Silicone elastomer	Pneumatic	~ 1.4	No	1	[14]
Silicone elastomer	Silicone elastomer	Pneumatic	0.67	No	1	[15]
Spring	Silicone elastomer	Pneumatic	3.2	No	1	[16]
Polyester ribbon	Silicone elastomer	Pneumatic	1.3	Yes	2	[17]
Soft polymer	Soft polymer	SMP	~ 0.1	No	1	[18]
Urethane elastomer	Urethane elastomer	SMA	1	No	1	[19]
Silicone elastomer	Silicone elastomer	SMA	0.5	Yes	1	This work

Supporting Movies

Movie S1: Multistable Actuator. In this video, we demonstrate the actuator's transition between all six stable states.

Movie S2: State Transitions at 0.5 Hz Frequency. In this video, the actuator performs 5 cycles of state transitions between two pure-bend stable states B1 and B2.

Movie S3: Motion Capture. In this video, we demonstrate the process of motion capturing, as well as compare it to a modeled result generated from the mocap data.

Movie S4: Visual Inspection with a Tip-mounted Camera. In this video, a demonstration based on a multistable actuator for visually inspecting is presented, with a tip-mounted miniature camera capturing information from a confined space, forming a string "SML2023".

Movie S5: Heliotropism-inspired Energy Harvesting. In this video, we highlight the potential application of the multistable actuator to improve the energy efficiency of a photovoltaic cell via employing multiple stable states.

Movie S6: Dexterously-turning Crawler. In this video, we demonstrate a single-actuator-based dexterously-turning crawler capable of both turning directions and crawling forward to move along a z-shaped zig-zag pathway.

Movie S7: Passive Multistable Structure. In this video, nitinol SMA coils are replaced by 3D-printed elastic rods, forming a fully passive multistable structure.

References

[1] A. A. Shabana, R. Y. Yakoub, *Journal of Mechanical Design, Transactions of the ASME* **2001**, *123*, 4 606.

[2] O. N. Dmitrochenko, D. Y. Pogorelov, *Multibody System Dynamics* **2003**, *10*, 1 17.

[3] O. N. Dmitrochenko, B. A. Hussein, A. A. Shabana, *Journal of Computational and Nonlinear Dynamics* **2009**, *4*, 2.

[4] X. Huang, J. Zou, G. Gu, *IEEE/ASME Transactions on Mechatronics* **2021**, *26*, 6 3175.

[5] X. Huang, X. Zhu, G. Gu, *IEEE Transactions on Robotics* **2022**, *38*, 6 3792.

[6] O. H. Yeoh, *Rubber Chemistry and Technology* **1993**, *66*, 5 754.

[7] V. Ramachandran, M. D. Bartlett, J. Wissman, C. Majidi, *Extreme Mechanics Letters* **2016**, *9* 282.

[8] E. Loukaides, S. Smoukov, K. Seffen, *International Journal of Smart and Nano Materials* **2014**, *5*, 4 270.

[9] L. S. Novelino, Q. Ze, S. Wu, G. H. Paulino, R. Zhao, *Proceedings of the National Academy of Sciences* **2020**, *117*, 39 24096.

[10] S. Wu, Q. Ze, J. Dai, N. Udipi, G. H. Paulino, R. Zhao, *Proceedings of the National Academy of Sciences* **2021**, *118*, 36 e2110023118.

[11] A. H. Gelebart, D. Jan Mulder, M. Varga, A. Konya, G. Vantomme, E. W. Meijer, R. L. Selinger, D. J. Broer, *Nature* **2017**, *546*, 7660 632.

[12] H. Shao, S. Wei, X. Jiang, D. P. Holmes, T. K. Ghosh, *Advanced Functional Materials* **2018**, *28*, 35 1802999.

[13] M. Follador, A. T. Conn, J. Rossiter, *Smart Materials and Structures* **2015**, *24*, 6 065037.

[14] P. Rothemund, A. Ainla, L. Belding, D. J. Preston, S. Kurihara, Z. Suo, G. M. Whitesides, *Science Robotics* **2018**, *3*, 16 eaar7986.

[15] Y. Chi, Y. Tang, H. Liu, J. Yin, *Advanced Materials Technologies* **2020**, *5*, 9 2000370.

[16] Y. Tang, Y. Chi, J. Sun, T.-H. Huang, O. H. Maghsoudi, A. Spence, J. Zhao, H. Su, J. Yin, *Science Advances* **2020**, *6*, 19 eaaz6912.

[17] Y. Chi, Y. Hong, Y. Zhao, Y. Li, J. Yin, *Science Advances* **2022**, *8*, 46 eadd3788.

[18] T. Chen, O. R. Bilal, K. Shea, C. Daraio, *Proceedings of the National Academy of Sciences* **2018**, *115*, 22 5698.

[19] D. K. Patel, X. Huang, Y. Luo, M. Mungekar, M. K. Jawed, L. Yao, C. Majidi, *Advanced Materials Technologies* **2023**, *8*, 2 2201259.
